# Supplementary material for: Protein Stability Buffers the Cost of Translation Attenuation following eIF2α Phosphorylation
Source: Cell Rep. 2020 Sep 15;32(11):108154. doi: 10.1016/j.celrep.2020.108154 (PMC7495045; doi:10.1016/j.celrep.2020.108154)
Supplement: Document S1. Tables S1 and S2 and Figures S1–S9 [file mmc1.pdf]

Cell Reports, Volume *32*

Supplemental Information

Protein Stability Buffers the Cost of Translation Attenuation following eIF2α Phosphorylation

Kim Schneider, Geoffrey Michael Nelson, Joseph Luke Watson, Jörg Morf, Maximillian Dalglish, Laura Martina Luh, Annika Weber, and Anne Bertolotti

Supplemental Information

Table S1: Top 10 GO terms of translationally regulated mRNAs as determined by anota2seq, Related to Figure 1

Top 10 GO terms of relative translationally down- or upregulated mRNAs as determined by anota2seq at 2 and 4 hours following Tm injection.

Translationally downregulated mRNAs at 2 hours

Category	GO terms	count	%	p value	**adjusted p value**
GOTERM_BP_DIRECT	translation	136	8.2	1.90E-52	6.60E-49
GOTERM_BP_DIRECT	transport	232	14	2.50E-15	4.50E-12
GOTERM_BP_DIRECT	mitochondrial translation	21	1.3	1.00E-13	1.20E-10
GOTERM_BP_DIRECT	protein transport	99	6	2.40E-13	2.20E-10
GOTERM_BP_DIRECT	rRNA processing	36	2.2	1.10E-11	8.10E-09
GOTERM_BP_DIRECT	oxidation-reduction process	101	6.1	1.20E-10	7.30E-08
GOTERM_BP_DIRECT	glutathione metabolic process	21	1.3	1.50E-10	7.40E-08
GOTERM_BP_DIRECT	antigen processing and presentation of exogenous peptide antigen via MHC class I, TAP-dependent	16	1	9.10E-10	4.00E-07
GOTERM_BP_DIRECT	proton transport	21	1.3	4.90E-09	1.90E-06
GOTERM_BP_DIRECT	vesicle-mediated transport	43	2.6	1.20E-08	4.20E-06

Translationally downregulated mRNAs at 4 hours

Category	GO terms	count	%	p value	**adjusted p value**
GOTERM_BP_DIRECT	translation	105	10.	7.20E-50	1.80E-46
GOTERM_BP_DIRECT	transport	144	14.	4.90E-11	6.10E-08
GOTERM_BP_DIRECT	oxidation-reduction process	69	6.9	8.00E-10	6.60E-07
GOTERM_BP_DIRECT	proton transport	17	1.7	4.20E-09	2.60E-06
GOTERM_BP_DIRECT	ATP synthesis coupled proton	11	1.1	2.50E-08	1.30E-05
GOTERM_BP_DIRECT	ribosomal small subunit assembly	10	1	1.50E-07	6.20E-05
GOTERM_BP_DIRECT	cytoplasmic translation	12	1.2	1.90E-07	6.60E-05
GOTERM_BP_DIRECT	mitochondrial translation	12	1.2	3.60E-07	1.10E-04
GOTERM_BP_DIRECT	rRNA processing	20	2	4.50E-06	1.20E-03
GOTERM_BP_DIRECT	ATP metabolic process	11	1.1	9.20E-06	2.30E-03

Translationally upregulated mRNAs at 2 hours

Category	GO terms	count	%	p value	**adjusted p value**
GOTERM_BP_DIRECT	mRNA processing	95	5.1	3.50E-25	1.60E-21
GOTERM_BP_DIRECT	metabolic process	116	6.2	6.20E-24	1.50E-20
GOTERM_BP_DIRECT	RNA splicing	70	3.8	2.40E-18	3.80E-15
GOTERM_BP_DIRECT	protein transport	110	5.9	1.00E-12	1.20E-09
GOTERM_BP_DIRECT	covalent chromatin modification	61	3.3	4.10E-11	3.80E-08
GOTERM_BP_DIRECT	transcription, DNA-templated	255	13.	6.70E-11	5.30E-08
GOTERM_BP_DIRECT	flavonoid biosynthetic process	15	0.8	1.80E-10	1.20E-07

GOTERM_BP_DIRECT	flavonoid glucuronidation	15	0.8	1.80E-10	1.20E-07
GOTERM_BP_DIRECT	oxidation-reduction process	113	6.1	4.30E-10	2.50E-07
GOTERM_BP_DIRECT	DNA repair	65	3.5	1.40E-09	7.40E-07

Translationally upregulated mRNAs at 4 hours

Category	GO terms	count	%	p value	**adjusted p value**
GOTERM_BP_DIRECT	flavonoid glucuronidation	12	1.8	6.20E-12	1.40E-08
GOTERM_BP_DIRECT	flavonoid biosynthetic process	12	1.8	6.20E-12	1.40E-08
GOTERM_BP_DIRECT	xenobiotic glucuronidation	9	1.4	7.20E-12	8.20E-09
GOTERM_BP_DIRECT	metabolic process	38	5.7	1.50E-07	1.20E-04
GOTERM_BP_DIRECT	transcription, DNA-templated	93	14	6.90E-06	3.90E-03
GOTERM_BP_DIRECT	mRNA processing	25	3.8	7.20E-05	3.20E-02
GOTERM_BP_DIRECT	protein ubiquitination involved in ubiquitin-dependent protein catabolic process	15	2.3	7.30E-05	2.80E-02
GOTERM_BP_DIRECT	regulation of transcription, DNA-	103	15.	7.40E-05	2.40E-02
GOTERM_BP_DIRECT	response to nutrient	10	1.5	4.10E-04	1.10E-01
GOTERM_BP_DIRECT	protein ubiquitination	25	3.8	4.20E-04	1.00E-01

Table S2: Oligonucleotides used in this study

*MmPpp1r15a*_R: tctcaggtcctccttcctca	Sigma-Aldrich	N/A
*MmChop*_F: cctagcttggctgacagagg	Sigma-Aldrich	N/A
*MmChop*_R: ctgctccttctccttcatgc	Sigma-Aldrich	N/A
*MmBiP*_F: ctttgatcagcgggtcatgg	Sigma-Aldrich	N/A
*MmBiP*_R: agctcttcaaatttggcccg	Sigma-Aldrich	N/A
*MmGrp94*_F: agtcgggaagcaacagagaa	Sigma-Aldrich	N/A
*MmGrp94*_R: tctccatgttgccagaccat	Sigma-Aldrich	N/A
*MmNedd4*_F: atgtggatgctgggagttga	Sigma-Aldrich	N/A
*MmNedd4*_R: ctgcacgctggtaaggattc	Sigma-Aldrich	N/A
*MmRps6*_F: aaggtaagaagcccaggacc	Sigma-Aldrich	N/A
*MmRps6*_R: ctgttcctggcgcttttctt	Sigma-Aldrich	N/A
*MmCul4b*_F: cccttgtcttccttgcacac	Sigma-Aldrich	N/A
*MmCul4b*_R: aggaagcaaagcagagtcct	Sigma-Aldrich	N/A
*MmAsns*_F: aggaggcatcgagaaaacca	Sigma-Aldrich	N/A
*MmAsns*_R: aagaagggagtggtggagtg	Sigma-Aldrich	N/A
HsRpl4_F: tgggacgtttctgcatttgg	Sigma-Aldrich	N/A
HsRpl4_R: cgatggatcttcttgcgtgg	Sigma-Aldrich	N/A
HsMrpl12_F: ggaggcggtggaagaagata	Sigma-Aldrich	N/A
HsMrpl12_R: gactccaccagcttctttgc	Sigma-Aldrich	N/A
HsMrpl28_F: ggacctgtgctccaagtttg	Sigma-Aldrich	N/A
HsMrpl28_R: ttgaacaggggtacagggtc	Sigma-Aldrich	N/A
HsMrps15_F: ggccaacaagaaggagatgc	Sigma-Aldrich	N/A
HsMrps15_R: ttgtgggctttgtcctttcg	Sigma-Aldrich	N/A
HsMrps25_F: cagggaagaggaggaggaga	Sigma-Aldrich	N/A
HsMrps25_R: aacccttgggtgcagatcat	Sigma-Aldrich	N/A
MmMrpl12_F: tcctcctcctcttccctgag	Sigma-Aldrich	N/A
MmMrpl12_R: cctccaaggctgctttgatc	Sigma-Aldrich	N/A
MmMrpl28_F: ccccggtacactacaaacct	Sigma-Aldrich	N/A
MmMrpl28_R: tccacaccttcttcaccctc	Sigma-Aldrich	N/A
MmMrps15_F: caagcaggacgatgaaccac	Sigma-Aldrich	N/A
MmMrps15_R: attgcggatcctgacagtca	Sigma-Aldrich	N/A
MmMrps25_F: cccttcctgcggttctatct	Sigma-Aldrich	N/A

MmMrps25_R: ggccttctacttcgcacatg	Sigma-Aldrich	N/A
*Cyclophilin B*_F: ggagatggcacaggaggaa	Sigma-Aldrich	N/A
*Cyclophilin B*_R: gcccgtagtgcttcagctt	Sigma-Aldrich	N/A
*Luciferase*_F: aaaggatatcaggtggcccc	Sigma-Aldrich	N/A
*Luciferase*_R: ccacaaacacaactcctccg	Sigma-Aldrich	N/A
*Atp5d*_5UTR_F: gatacaactagtccttcggagaatcctgtgc	Sigma-Aldrich	N/A
*Atp5d*_5UTR_R: gatacaccatggggtggcggaaaagtcagtg	Sigma-Aldrich	N/A
*Atf4*_5UTR_F: gatacaactagtggctaggtgtcccaccc	Sigma-Aldrich	N/A
*Atf4*_5UTR_R: gatacagaattcgttgtggggctttgctggattcg	Sigma-Aldrich	N/A
*Mrps28*_5UTR_F: gatacagaattcaggcaagctttaaactaccggac	Sigma-Aldrich	N/A
*Mrps28*_5UTR_R: gatacaccatgggacgcctgcgcgacctc	Sigma-Aldrich	N/A
*Mrps33*_5UTR_F: gatacaactagttccgggttgtccgccgac	Sigma-Aldrich	N/A
*Mrps33*_5UTR_R: gatacaccatggttcttcaccagtagggaacaggattcc	Sigma-Aldrich	N/A
*Rps15a*_5UTR_F: gatacaactagtataattccgtgcgtttgcccggcccactctctttctgccatcttccctcgccgccacc	Sigma-Aldrich	N/A
*Rps15a*_5UTR_R: gatacaccatggggtggcggcgagggaagatggcagaaagagagtgggccgggcaaacgcacggaattat	Sigma-Aldrich	N/A
*Rps18*_5UTR_F: gatacaactagtgtcacttccgctctctcttccacaggaggcctacacgccgccgtctgtgccgccgcc	Sigma-Aldrich	N/A
*Rps18*_5UTR_R: gatacaccatggggcggcggcacagacggcggcgtgtaggcctcctgtggaagagagagcggaagtgac	Sigma-Aldrich	N/A
*Rpl35*_5UTR_F: ggactagtgaacagtggaagaagagaactc	Sigma-Aldrich	N/A
*Rpl35*_5UTR_F: catgccatggttcggcgttgttcacg	Sigma-Aldrich	N/A
*Rps24*_5UTR_F: ggactagtgctggggtccttcctt	Sigma-Aldrich	N/A
*Rps24*_5UTR_R: catgccatgggatggctacggcgcc	Sigma-Aldrich	N/A
*Rps15*_5UTR_F: gatacaactagtgtacttccttttccgagtaaccgccaag	Sigma-Aldrich	N/A
*Rps15*_5UTR_R: gatacaccatggcttggcggttactcggaaaaggaagtac	Sigma-Aldrich	N/A

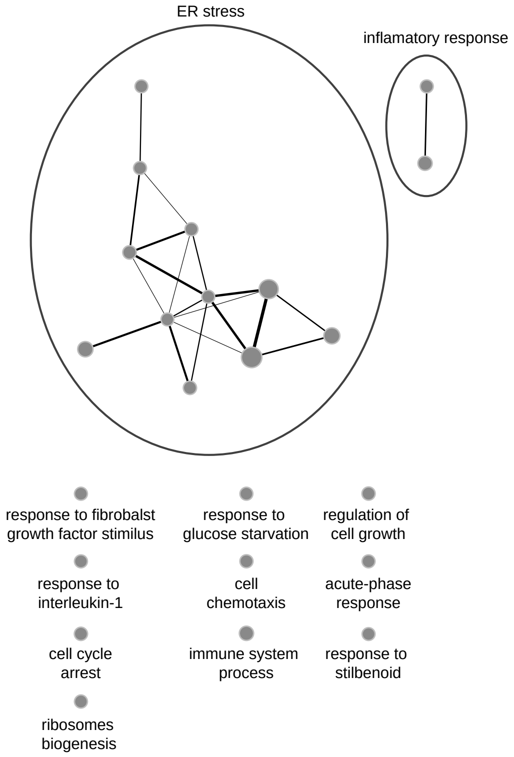

Figure S1. Gene ontology enrichment map of mRNAs transcriptionally upregulated after Tm, Related to Figure 1

Significant (P<0.001) gene ontology enrichment maps of the 900 mRNAs transcriptionally upregulated (2-fold cut-off) 4 hours after Tm injection.

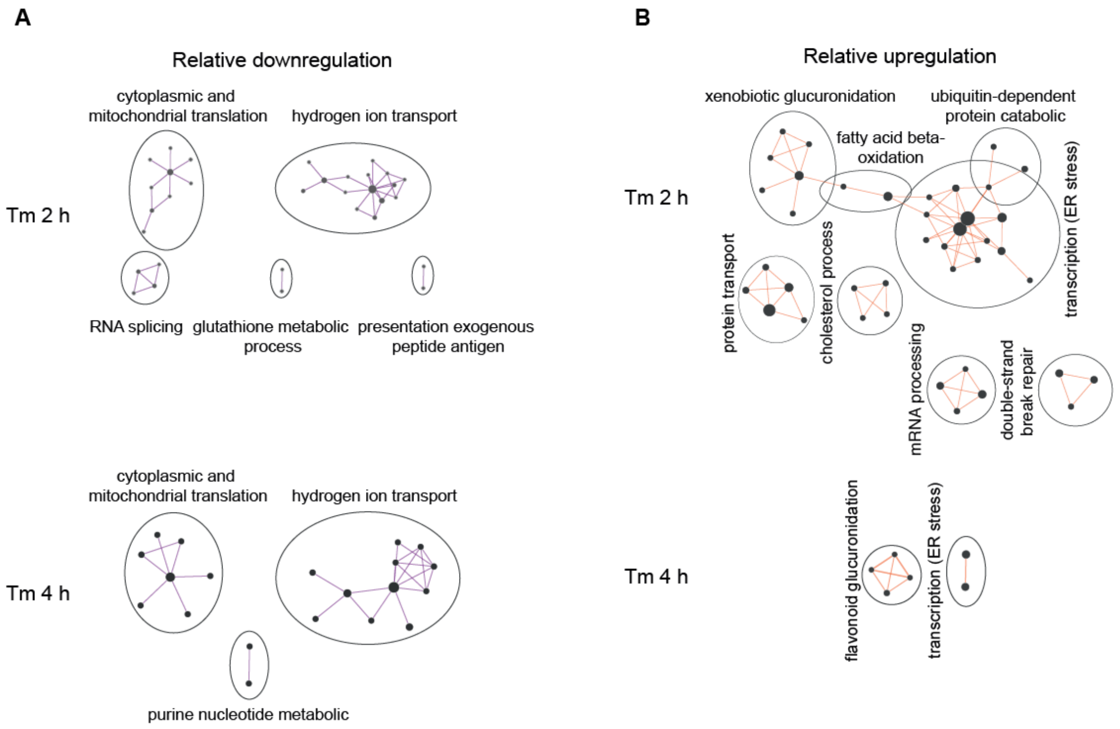

Figure S2. GO analysis of mRNA translationally affected following Tm, Related to Figure 3

(A) Significant (P<0.001) GO enrichment maps of translationally downregulated mRNAs as determined by anota2seq at 2 and 4 hours following Tm injection.

(B) Same as (A) for genes resistant to translational attenuation (relative upregulation).

A

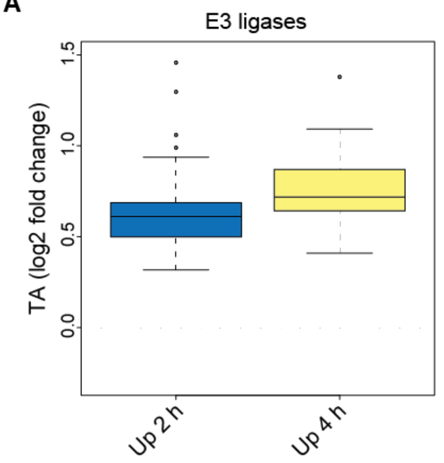

B

E3 ligases upregulated (relative upregulation) at 2 h

Klhl9	*Ubr2*	*Fbxo11*	*Brca1*
Klhl28	*Trip12*	*Usp36*	*G2E3*
Klhdc7A	*Bag6*	*Trim23*	*Usp34*
Cdc73	*Ddb1*	*Ubr3*	*Fbxo21*
Pja2	*Dzip3*	*Rnf111*	*Rnf123*
Klhl22	*Usp24*	*Fbxo10*	*Usp33*
Cand1	*Asb3*	*Cyld*	*Usp22*
Usp4	*Nfe2L2*	*Usp9X*	*Dcaf8*
Shprh	*Abtb1*	*Dcaf17*	*Ddb2*
Usp47	*Tnks*	*Sash1*	*Usp48*
Leo1	*Usp19*	*Pdzrn3*	
Ltn1	*Hectd3*	*Kbtbd4*	
Uvssa	*Pja1*	*Ctr9*	
Hltf	*Trim33*	*Cdc20*	
Med17	*Sqstm1*	*Ttc3*	

E3 ligases upregulated (relative upregulation) at 4 h

Klhl28	*Nedd4*
Crbn	*Usp47*
Socs4	*Ubr5*
Klhl24	*Arntl*
Vps4B	*Fbxo21*
Klhl22	*Fbxo30*
Cul4B	*Ube3A*
Rnf216	*Mib2*
Vps37A	*Asb3*
Klhdc7A	*Cbll1*
Rnf125	
Usp34	
Ubr7	
Cyld	
Lrrc41	

Figure S3. Relative translational regulation of E3 ligases following Tm, Related to Figure 3

(A) Boxplot showing the median translation efficiency (TA) as determined by anota2seq at 2 and 4 hours following Tm treatment.

(B) List of E3 ligases translationally upregulated (relative upregulation) at 2 and 4 hours following Tm, respectively.

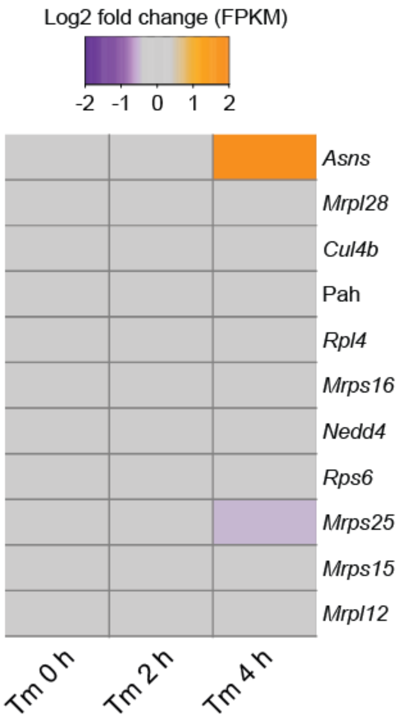

Figure S4. mRNAs abundance of selected transcripts, Related to Figure 3
Log2 FPKM values for indicated genes at 0, 2 and 4 hours following Tm.

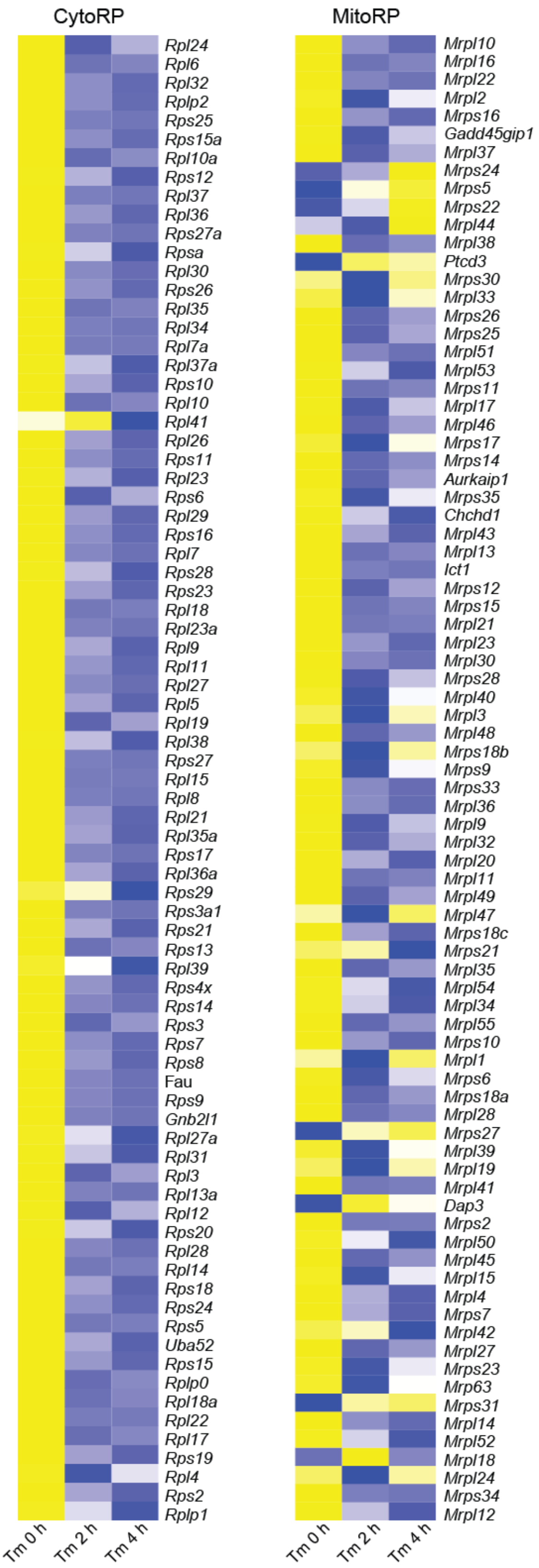

Figure S5. Cytosolic and mitochondrial ribosomal proteins are translationally downregulated by Tm, Related to Figure 3

(A) Heat map showing z-scores of anota2seq translation efficiencies of all 79 cytosolic ribosomal proteins (CytoRP) subunit-encoding mRNAs at 0, 2 and 4 hours following Tm injection.

(B) Same as (A) but with all 82 mitochondrial ribosomal proteins (MitoRP) subunits.

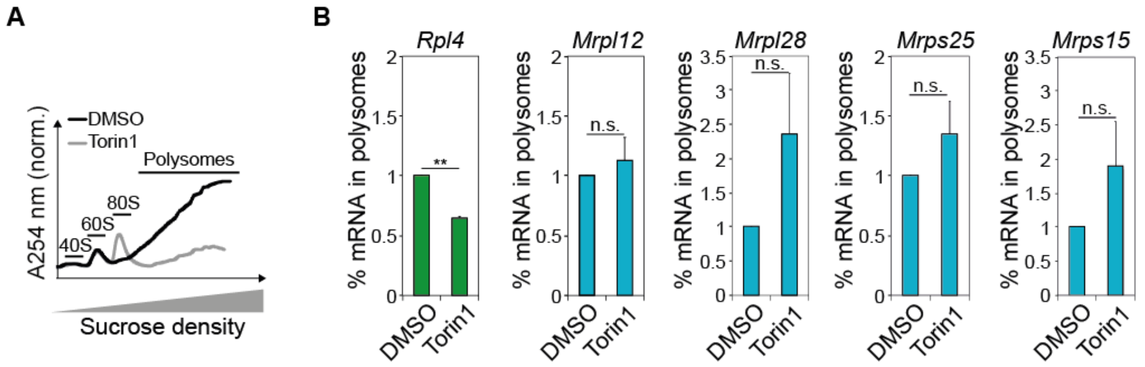

Figure S6. Translation of mitochondrial ribosomal proteins is not sensitive to acute mTORC1 inhibition, Related to Figure 4

(A) Representative polysome profile (optical density of the fractions at 254 nm) on 5-50% sucrose gradients of HeLa cells treated with DMSO or 250 nM of Torin1 for 2 hours.

(B) *Rpl4*, *Mrpl12*, *Mrpl28, Mrps25 and Mrps15* mRNA abundance from heavy polysomal fractions (more than 3 ribosomes) from (A) assessed by qPCR.

Data are means ± SEM (*n* = 5-9). **P<0.01 as determined by the unpaired Student t-test.

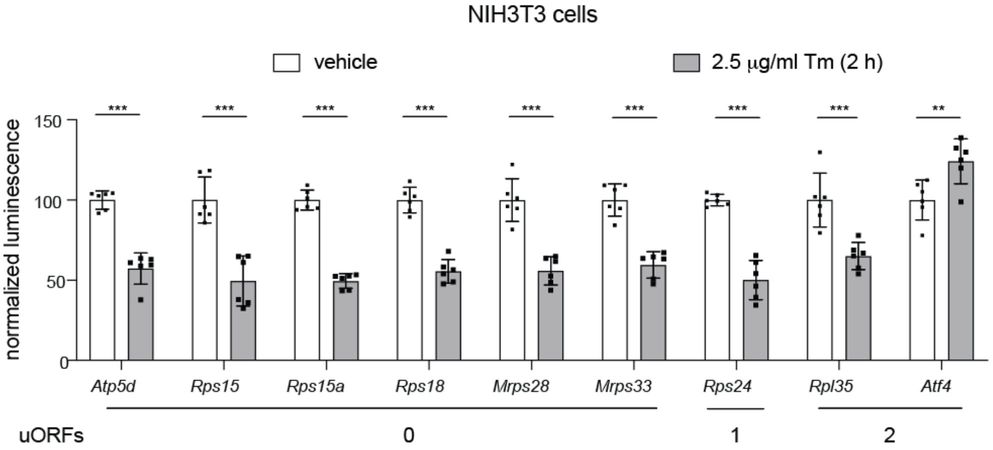

Figure S7. Composition of UTRs regulates the translational response to eIF2α phosphorylation, Related to Figure 5

Firefly luciferase activity in NIH3T3 cells transfected with reporter mRNAs containing the 5′UTRs of indicated mRNAs and treated with either vehicle or 2.5 μg/ml Tunicamycin (Tm) for 2 hours. Firefly luciferase activity was normalized against a transfection control (see Methods) and vehicle-treated cells. Data are means ± SEM (n=6). Statistical analysis was carried out in Prism 8 using a two-way ANOVA test.

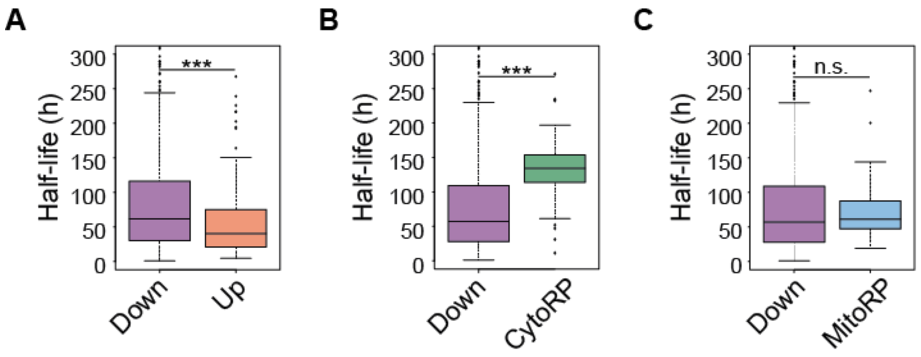

Figure S8. Translationally downregulated mRNAs encode for highly stable proteins, Related to Figure 6

Same as in Figure 6E with protein half-lives analyzed from (Schwanhäusser et al, 2013).

(A) Boxplot of half-lives of proteins encoded by down- or upregulated (Down or Up) mRNAs at 2 hours following Tm injection.

(B) Same as (A) comparing the half-lives of proteins encoded by translationally downregulated mRNAs with all 79 CytoRP subunits 2 hours after Tm.

(C) Same as (B) with all 82 MitoRP subunits. (A-C) ***P<0.001 as determined by the unpaired Wilcoxon test.

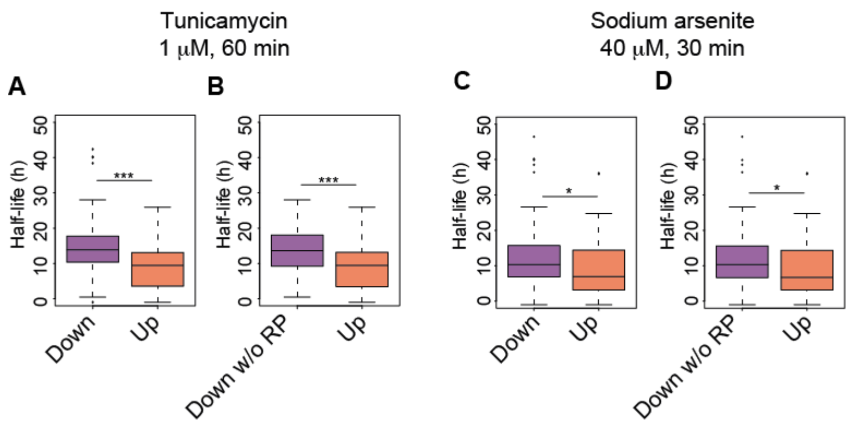

Figure S9. eIF2α phosphorylation induced by different stressors downregulates translation of mRNAs encoding for stable proteins, Related to Figure 6

(A) Boxplot of half-lives of proteins encoded by the translationally down or upregulated (Down or Up) mRNAs using data from (Sidrauski et al, 2015) 1 hours following Tm treatment of HEK293T cells.

(B) Same as (A) but excluding the RPs from the category of translationally downregulated mRNAs (Down w/o RP).

(C) Same as (A) but with data from (Andreev et al, 2015) acquired 30 minutes following arsenite treatment of HEK293T cells.

(D) Same as (C) but excluding the RPs from the category of translationally downregulated mRNAs (Down w/o RP).

(A-D) Protein half-lives were analyzed from (Ly et al, 2018)

*$P<0.05$, ***$P<0.001$ as determined by the unpaired Wilcoxon test.
