# Supplementary material for: Protein Stability Buffers the Cost of Translation Attenuation following eIF2α Phosphorylation
Source: Cell Rep. 2020 Sep 15;32(11):108154. doi: 10.1016/j.celrep.2020.108154 (PMC7495045; doi:10.1016/j.celrep.2020.108154)
Supplement: Document S2. Article plus Supplemental Information [file mmc2.pdf]

Cell Reports

Article

Protein Stability Buffers the Cost of Translation Attenuation following eIF2α Phosphorylation

Graphical Abstract

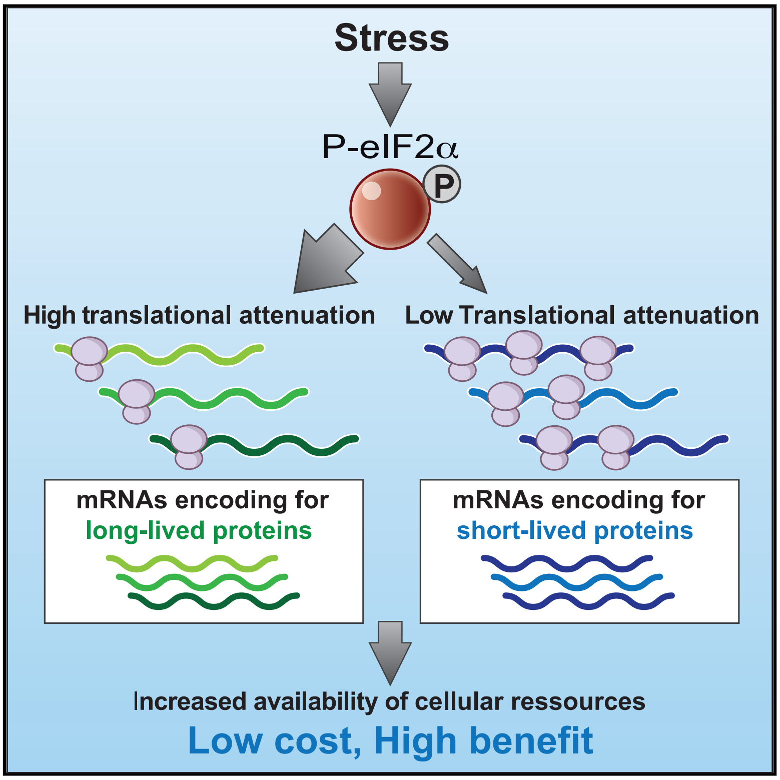

Authors

Kim Schneider, Geoffrey Michael Nelson, Joseph Luke Watson, ..., Laura Martina Luh, Annika Weber, Anne Bertolotti

Correspondence

kim.sarah.schneider1@gmail.com (K.S.), aberto@mrc-lmb.cam.ac.uk (A.B.)

In Brief

Here, we find that translation attenuation following eIF2α phosphorylation is not uniform, with long-lived proteins, including cytosolic and mitochondrial ribosomal subunits, being prominently downregulated. Because protein stability buffers the cost of translational attenuation, there is no measurable decrease of the targeted proteins. This reveals an evolutionary principle of cellular robustness.

Highlights

- Translation attenuation following eIF2α phosphorylation is not uniform
- Low translation of downregulated targets persists after global translation recovery
- Translation attenuation following eIF2α phosphorylation favors long-lived proteins
- Protein stability buffers the cost of translational attenuation

Schneider et al., 2020, Cell Reports *32*, 108154
September 15, 2020 © 2020 MRC Laboratory of Molecular Biology.
https://doi.org/10.1016/j.celrep.2020.108154

Cell Reports

CellPress OPEN ACCESS

Article

Protein Stability Buffers the Cost of Translation Attenuation following eIF2α Phosphorylation

Kim Schneider,[1,*] Geoffrey Michael Nelson,[1,3,4] Joseph Luke Watson,[1,3] Jörg Morf,[2] Maximillian Dalglish,[1] Laura Martina Luh,[1] Annika Weber,[1] and Anne Bertolotti[1,5,*]

[1]MRC Laboratory of Molecular Biology, Francis Crick Avenue, Cambridge CB2 0QH, United Kingdom

[2]Wellcome - MRC Cambridge Stem Cell Institute, Puddicombe Way, Cambridge CB2 0AW, United Kingdom

[3]These authors contributed equally

[4]Present address: Department of Biomedical Informatics, Harvard Medical School, Boston, MA, USA

[5]Lead Contact

*Correspondence: kim.sarah.schneider1@gmail.com (K.S.), aberto@mrc-lmb.cam.ac.uk (A.B.)

https://doi.org/10.1016/j.celrep.2020.108154

SUMMARY

Phosphorylation of the translation initiation factor eIF2α is a rapid and vital response to many forms of stress, including protein-misfolding stress in the endoplasmic reticulum (ER stress). It is believed to cause a general reduction in protein synthesis while enabling translation of few transcripts. Such a reduction of protein synthesis comes with the threat of depleting essential proteins, a risk thought to be mitigated by its transient nature. Here, we find that translation attenuation is not uniform, with cytosolic and mitochondrial ribosomal subunits being prominently downregulated. Translation attenuation of these targets persists after translation recovery. Surprisingly, this occurs without a measurable decrease in ribosomal proteins. Explaining this conundrum, translation attenuation preferentially targets long-lived proteins, a finding not only demonstrated by ribosomal proteins but also observed at a global level. This shows that protein stability buffers the cost of translational attenuation, establishing an evolutionary principle of cellular robustness.

INTRODUCTION

Organisms have evolved adaptive mechanisms to survive abrupt changes in their environment. Phosphorylation of the translation initiation factor eIF2α is a rapid and vital response to many forms of stress, including protein-misfolding stress in the endoplasmic reticulum (ER stress) (Harding et al., 2002; Hinnebusch et al., 2016; Pilla et al., 2017; Wek, 2018). It causes a reduction in protein synthesis while enabling translation of few transcripts (Young and Wek, 2016).

eIF2α, eIF2β, and eIF2γ are subunits of the essential trimeric translation initiation factor, eIF2. eIF2-guanosine triphosphate (GTP) brings initiator methionyl tRNA to the 40S ribosomal subunit and other initiation factors to form the 43S preinitiation complex (PIC). The PIC is recruited to the 5′ methylguanine cap of an mRNA and scans the 5′ UTR for an AUG initiation codon (Sonenberg and Hinnebusch, 2009). Following start codon recognition, GTP is hydrolyzed, releasing phosphate and eIF2-guanosine diphosphate (GDP) and allowing binding of the 60S ribosomal subunit and translation elongation to proceed. eIF2-GDP needs to be reactivated into eIF2-GTP to participate in another round of translation initiation, a reaction catalyzed by the guanine nucleotide exchange factor eIF2B. Phosphorylation of eIF2α inhibits eIF2B, thereby reducing translation initiation (Krishnamoorthy et al., 2001). Because the eIF2B complex is limiting in cells relative to eIF2, a small amount of phosphorylated eIF2α has profound consequences (Young and Wek, 2016).

eIF2α phosphorylation is an essential, evolutionarily conserved signaling event: Yeast or mammalian cells harboring a non-phosphorylable eIF2α die in the presence of stress (Dever et al., 1992; Pilla et al., 2017; Scheuner et al., 2001). Importantly, the number of eIF2α kinases and phosphatases has expanded with evolution (Pilla et al., 2017), indicating an evolutionary pressure to not only retain eIF2α phosphorylation but also increase and diversify its use. While yeast have only one eIF2α kinase (GCN2, activated by amino acid shortage), mammals have three additional eIF2α kinases sensing diverse signals: PKR, activated by viral infections; HRI, activated by heme deficiency; and PERK (or PEK), activated by misfolded proteins in the ER (Sonenberg and Hinnebusch, 2009). To maintain homeostatic levels of eIF2α phosphorylation, mammals have two eIF2α phosphatases that antagonize the four eIF2α kinases. These enzymes are split enzymes composed of a common catalytic subunit, the protein phosphatase 1 (PP1c), bound to one of two specific substrate receptors: The stress-inducible PPP1R15A (R15A), or the constitutive PPP1R15B (R15B) (Bertolotti, 2018). The holoenzymes R15A-PP1c and R15B-PP1c are selective for their substrates (Bertolotti, 2018; Carrara et al., 2017; Harding et al., 2009), with R15A-PP1c being particularly important following stress, to ensure rapid translation recovery (Novoa et al., 2003).

This is an open access article under the CC BY license (http://creativecommons.org/licenses/by/4.0/).

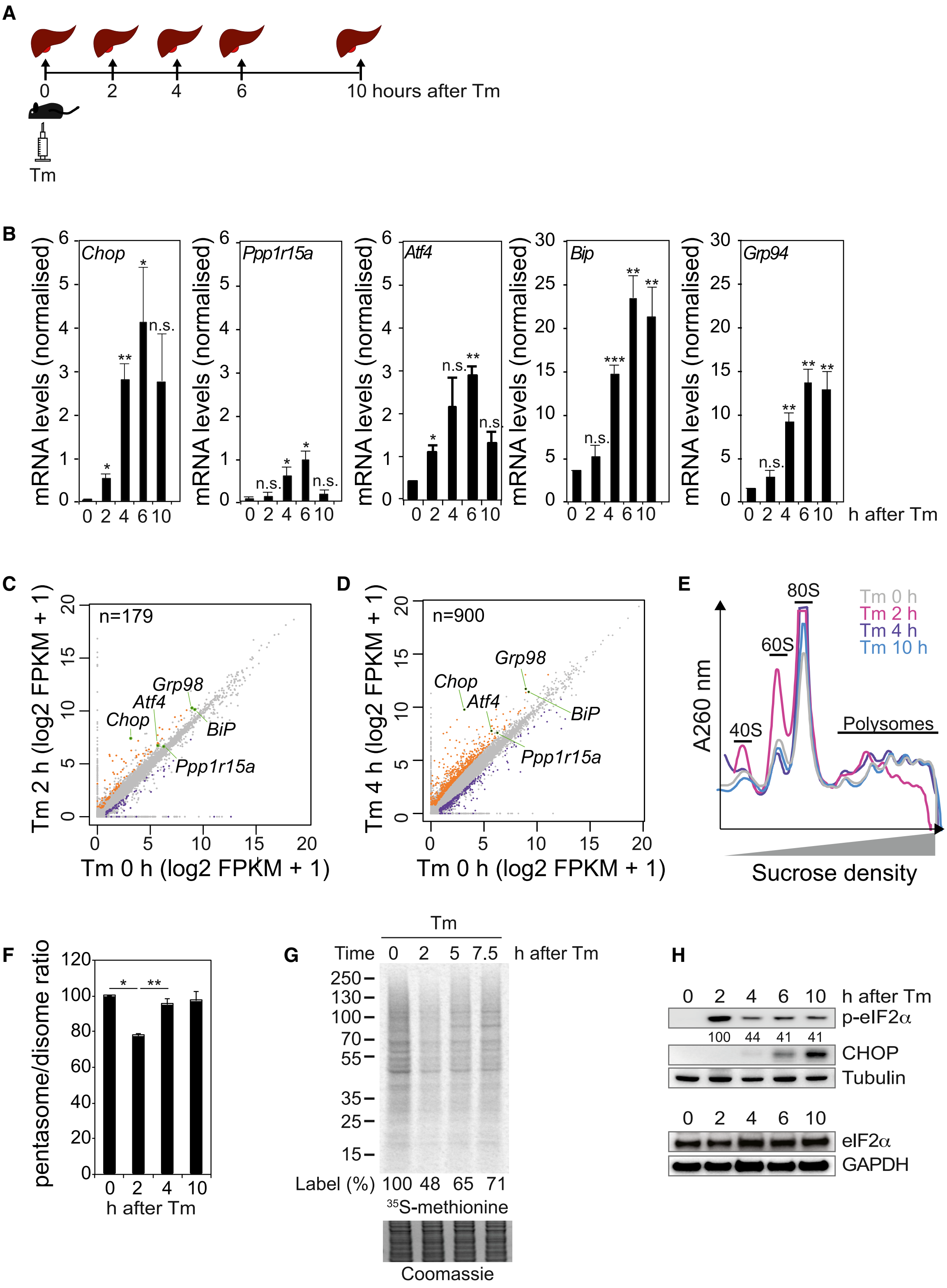

(legend on next page)

The consequences of eIF2α phosphorylation have been well characterized in the context of ER stress. Historically, this response has been monitored using metabolic labeling or polysome profiling (Brostrom and Brostrom, 1998; Brostrom et al., 1995, 1996; Wong et al., 1993). Because ER stress leads to a decreased incorporation of ^{35}S-methione and a reduction of polysomes, it has been concluded that eIF2α phosphorylation leads to a global decrease in protein synthesis (Brostrom and Brostrom, 1998; Brostrom et al., 1995, 1996; Wong et al., 1993). A few transcripts have been found to escape translation attenuation upon stress (Young and Wek, 2016). Recent in-depth analyses of the translational changes following eIF2α phosphorylation have revealed how translation recovery is coordinated with transcriptional reprogramming (Guan et al., 2017) and identified a few mRNAs selectively translated when eIF2α is phosphorylated (Sidrauski et al., 2015). However, the interpretation that eIF2α phosphorylation leads to a general, albeit transient, attenuation of translation, with a few exceptions, has been generally adopted since its initial discovery (Brostrom and Brostrom, 1998; Brostrom et al., 1995, 1996; Wong et al., 1993), but not formally examined.

In healthy organisms, a large fraction of the cellular resources is committed to protein synthesis (Buttgereit and Brand, 1995; Rolfe and Brown, 1997). Protein expression levels are thought to be evolutionarily optimized based on two opposing principles: the benefit of having a protein and the cost of making it (Dekel and Alon, 2005). According to this model, a general decrease in protein synthesis following eIF2α phosphorylation comes with the risk of reducing protein abundance below the minimum required to sustain cell viability. How such risk is mitigated is unclear, despite considerable knowledge of the eIF2α signaling pathway (Harding et al., 2002; Hinnebusch et al., 2016; Kaufman, 2004; Pilla et al., 2017; Wek, 2018). Here, we set out to answer this question, performing a global analysis of the translational response to eIF2α phosphorylation *in vivo* following ER stress.

We analyzed the acute response to tunicamycin (Tm) stress *in vivo* and found that translation attenuation is not uniform across all mRNAs but rather preferentially targets cytosolic and mitochondrial ribosomal proteins (RPs). In contrast to the cytosolic RPs that are translationally regulated by mTORC1 (Thoreen et al., 2012), the mitochondrial ribosomal subunits were insensitive to acute treatment with the selective mTORC1 inhibitor Torin1. The decreased translation of mitochondrial RPs upon ER stress was controlled by eIF2α phosphorylation and abrogated in cells unable to phosphorylate eIF2α. Surprisingly, eIF2α-mediated translation attenuation of mitochondrial RPs occurred without measurably decreasing mitochondrial translation or levels of RPs. This happens because translation attenuation preferentially targets long-lived proteins, a finding not only revealed by RPs but also observed at a global level. This work reveals that the cost of translational attenuation has been evolutionarily minimized by preferentially targeting highly stable proteins.

RESULTS

The UPR *In Vivo*

As eIF2α phosphorylation integrates diverse environmental cues, we set out to study this signaling event *in vivo* in a physiological context. We used Tm, which blocks N-linked glycosylation, to induce accumulation of misfolded proteins in the ER and analyzed this response in mouse liver (Oyadomari et al., 2008; Yamamoto et al., 2010) (Figure 1A). Tm triggers an ER unfolded protein response (UPR), which in mammals has both a translational component, entirely mediated by eIF2α phosphorylation and a transcriptional component, involving the IRE1 and ATF6 branches (Wang and Kaufman, 2016; Wiseman et al., 2010). As expected, Tm injection induced the expression of canonical ER-UPR transcripts such as *Chop*, *Bip*, *Ppp1r15a*, *Atf4*, and *Grp94*, as detected by quantitative PCR (qPCR) analyses and RNA sequencing (RNA-seq) of liver mRNAs (Figures 1B–1D and S1).

Having captured the transcription-wide baseline and stress-induced alterations, we next analyzed translational changes. Before stress, a typical polysome profile was observed with a clear separation of 40S, 60S, and 80S ribosomes and an abundance of polysomes in the heavy fractions, attesting high translation rates (Figures 1E and 1F). Attenuation of translation, manifested by a decrease in polysomes and an increase in monosomes (Figures 1E and 1F), was observed 2 h after injection of Tm. Translation attenuation was transient and recovery coincided with eIF2α dephosphorylation (Figures 1E–1H). The kinetics of translation attenuation and recovery was similar to that observed in cell culture (Figure 1G) and by Harding et al.

Figure 1. The Transcriptional and Translational UPR Landscape *In Vivo*

(A) Overview of the experimental design. Mice (n = 3) were injected intraperitoneally with 0.1 mg/kg tunicamycin (Tm), and livers were collected at 0, 2, 4, 6, and 10 h following injection.

(B) *Chop*, *Ppp1r15a*, *Atf4*, *Bip*, and *Grp94* mRNAs expression in mouse livers at the indicated times following Tm injection quantified by qPCR and normalized to cyclophilin. Data are mean ± SEM (n = 3). *$p < 0.05$, **$p < 0.01$, and ***$p < 0.001$, as determined by two-way ANOVA.

(C) FPKM values from RNA-seq showing the changes in transcript abundance in mouse liver 2 h following Tm injection. Downregulated (purple) and upregulated (orange) mRNAs that passed a 2-fold cut-off.

(D) Same as (C), but 4 h following Tm injection.

(E) Representative polysome profiles (optical density of the fractions at 260 nm) on 7%–47% sucrose gradients of mouse liver extracts collected after Tm injection for the indicated time.

(F) Quantification of polysome profiles such as shown in (E) and represented as a pentasomes (five ribosomes) to disomes (two ribosomes) ratio. Data are mean ± SEM (n = 3).

(G) Autoradiogram of [35S]-methionine-labeled proteins in cell lysates resolved by SDS-PAGE after a 10-min labeling pulse of HeLa cells exposed to Tm (2.5 μg/mL) for the indicated times. Lower panel is an image of the InstantBlue-stained gel.

(H) Representative immunoblots with indicated antibodies with lysates from mouse livers treated with Tm for the indicated time.

See also Figure S1 and Table S1.

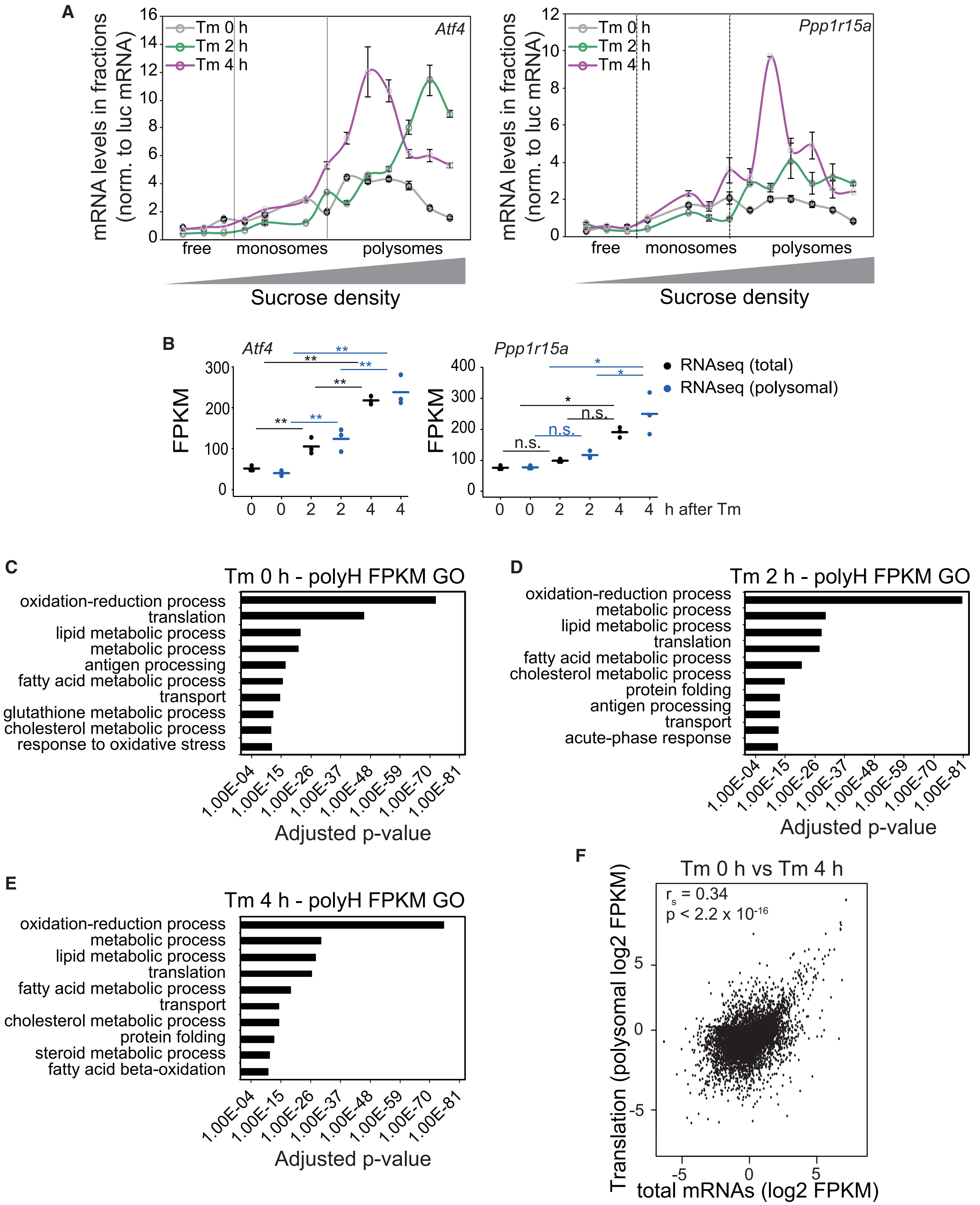

(legend on next page)

(2000b), with translation recovery concurring with production of stress proteins, such as CHOP, observed following eIF2α dephosphorylation (Figure 1H).

The translationally regulated ER stress mRNAs *Atf4* and *Ppp1r15a* were enriched in the polysomal fractions upon Tm treatment, as expected (Young and Wek, 2016), but this increase was associated with an increase in mRNA abundance (Figures 2A and 2B). The regulation of ATF4 has been so far described to be primarily translational (Harding et al., 2000a). Whilst *Atf4* mRNA was enriched in heavy polysome fractions after stress, as expected, indicating a high translation activity (Figure 2A), we were intrigued to observe a large increase in *Atf4* mRNA following stress (Figures 1B–1D and 2B). In fact, the transcriptional induction of *Atf4* was already evident in the first study linking ATF4 to stress (Harding et al., 2000a). However, it has remained so far uncharacterized, an interesting topic for future studies.

To gain insights into the nature of the translational changes, we then performed a polysome profiling analysis. This method was selected because it is believed to enable detection of a general decrease in translation of all mRNAs, as well as selective changes. Ribosome profiling has revolutionized studies in translation by revealing the activity of individual ribosomes (Ingolia et al., 2013). However, it is not believed to be an ideal method for assessing global decreases of translation (Ingolia et al., 2013). We thus performed a global analysis of polysome mRNA contents by RNA-seq of high-density polysomal fractions (containing more than three ribosomes) and ranked mRNA abundance under basal versus stress conditions. Gene Ontology (GO) enrichment analysis of the most abundant mRNAs in polysomal fractions identified two noticeable stress-induced changes: a decreased prominence of mRNAs involved in translation (Figures 2C–2E) and the appearance of mRNAs encoding protein folding in the top 10 list of GO terms (Figures 2C–2E). Overall, transcript abundance (total FPKM) and translation activity defined by FPKM value in heavy polysomes were partly correlated (Figure 2F).

Translation Attenuation by eIF2α Phosphorylation Is Not Uniform

To specifically identify relative stress-induced changes in translational efficiency without the confounding effect of transcriptional changes, we focused on the acute Tm response (before large transcriptional changes) and analyzed the RNA-seq results by anota2seq (analysis of translation activity), a software package designed to correct for changes in mRNA levels (Larsson et al., 2011; Oertlin et al., 2019). At 2 h, eIF2α phosphorylation was high (Figure 1H) and the ER stress response was largely translational (Figures 1C, 1E, and 3A), providing an ideal time point for this study. As previously reported, chronic stress involves coordination of transcriptional and translational reprogramming (Guan et al., 2017). The anota2seq analysis of the acute ER stress response revealed that 1,621 and 1,033 mRNAs were translationally downregulated at 2 and 4 h following Tm, respectively (Figures 3A and 3B). The anota2seq algorithm also identified subsets of "upregulated" mRNAs (1,924 at 2 h and 698 at 4 h following Tm), which represent the mRNAs most resistant to Tm-induced translational attenuation (Figures 3A and 3B). GO analysis of the downregulated mRNAs showed an enrichment of mRNAs encoding both cytoplasmic and mitochondrial translation components (Figure S2). In contrast, GO analysis of the mRNA resistant to Tm-induced translational downregulation revealed an enrichment for "ER stress," "metabolism-related functions," and "protein degradation" (Figure S2), with a high number of ubiquitin ligases (Figure S3). The persistence of translation of such mRNAs could contribute to resolving stress. The results of this analysis were validated for a subset of transcripts by qPCR of polysomal fractions (Figures 3C and 3D). We next examined mRNA abundance for these transcripts and found that their abundance was not measurably altered at 2 h after stress (Figure S4), confirming that these transcripts were translationally, but not transcriptionally, regulated at this time point. Thus, the global analyses of Tm-stress-induced changes presented here reveals that the translational downregulation following Tm is not as uniform as anticipated.

We next asked whether mRNAs encoding proteins translated at the ER were enriched in the downregulated group. An initial model rationalizing on the benefit of the PERK-mediated translation attenuation following ER stress proposed that one purpose of this pathway might be to reduce the load of clients (Ron and Harding, 2012). A proximity-specific ribosome profiling approach had previously identified proteins translated at the ER (Jan et al., 2014). We found that mRNAs encoding ER-translated proteins were not enriched in the pool of mRNAs downregulated by Tm (Figures 3E and 3F). This result demonstrates that although stress signaling was elicited in the ER, translation attenuation following eIF2α phosphorylation did not favor ER-translated proteins.

We next analyzed translation attenuation in more depth. 2 h after Tm, the most significant enriched GO category in the downregulated pool was "translation" (Table S1), including nearly all cytosolic and most mitochondrial RPs (Figure S5). Although cytosolic and mitochondrial RPs were prominently downregulated, they represented only ~10% of the downregulated proteins. While translation recovery manifested 2 h after stress (Figures 1E–1G), concomitantly to eIF2α dephosphorylation (Figure 1H), translation of the translationally downregulated mRNAs remained attenuated

Figure 2. Global Changes in Polysomal mRNAs following ER Stress

(A) *Atf4* and *Ppp1r15a* mRNA abundance across polysomal fractions at 2 and 4 h following Tm injection assessed by qPCR. Representative results of at least three independent experiments are shown. Data are mean ± SEM (n = 3).

(B) Total (RNA-seq) or polysomal (RNApoly) FPKM values for *Atf4* and *Ppp1r15a* at the indicated times after Tm injection. *p < 0.05 and **p < 0.001, as assessed by an unpaired Student's t test.

(C) Top 10 Gene Ontology terms ranked by adjusted p value of polysomal-associated transcripts (FPKM >50) before ER stress.

(D) Same as (C), but 2 h following Tm injection.

(E) Same as (C), but 4 h following Tm injection.

(F) Correlation between transcriptional changes (RNA-seq of total mRNAs, 2-fold cut-off) and polysome-association (RNA-seq of polysomal mRNAs) on the 900 Tm stress regulated mRNAs 4 h following Tm injection (r^s denotes Spearman correlation coefficient).

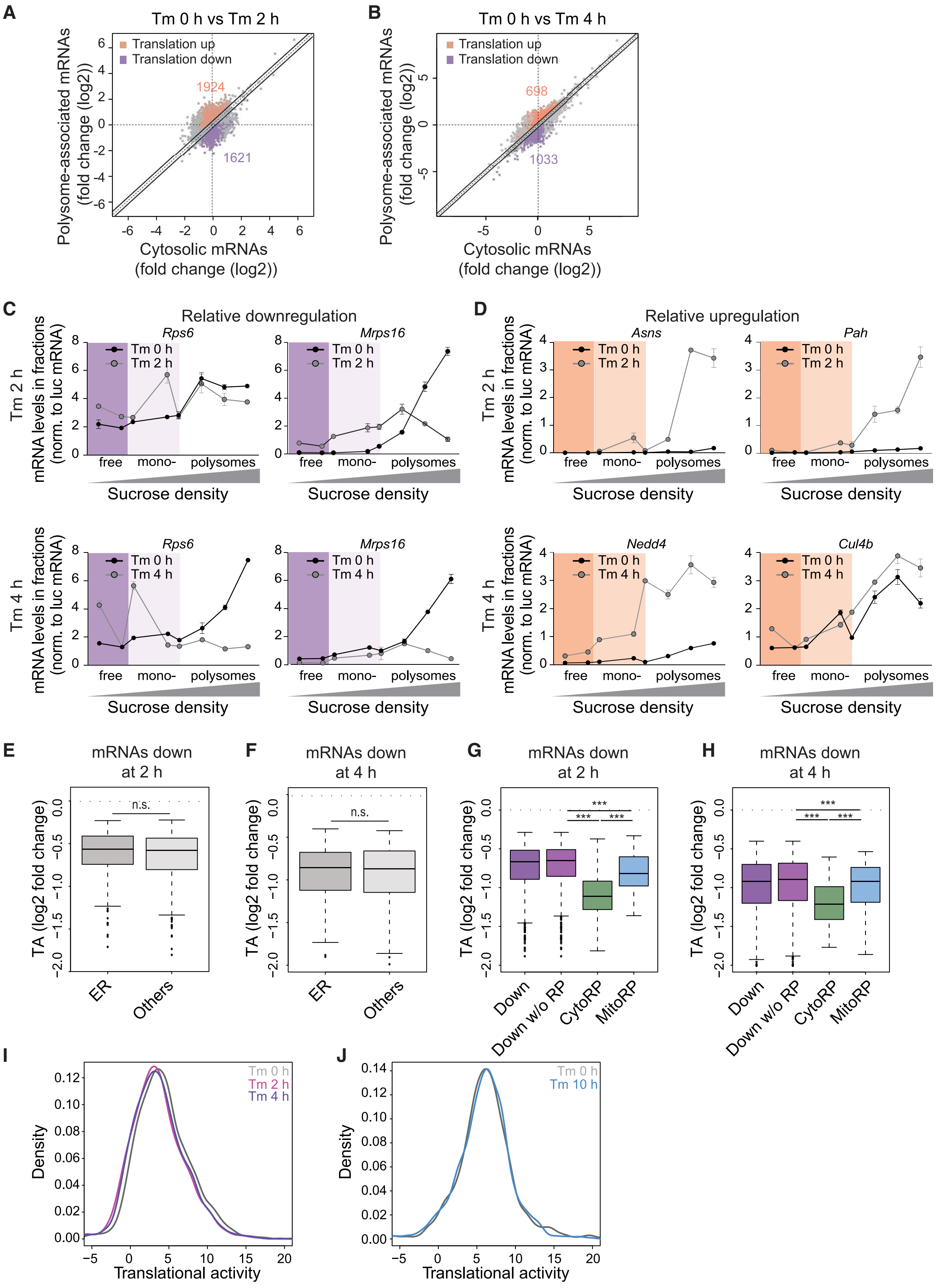

(legend on next page)

CellPress
OPEN ACCESS

at 4 h, with their recovery completed at 10 h following Tm (Figures 3G–3J and S5). This was surprising, because it demonstrates that the translationally downregulated targets remain so for longer than anticipated and also suggests that the recovery of global translation activity at 4 h was not mediated by increasing translation of the attenuated mRNAs but probably contributed by increased translation of stress genes, in line with other studies (Guan et al., 2017; Novoa et al., 2003). The finding that translation attenuation of eIF2α targets persists for a prolonged period of time, beyond the point where translation recovery is observed at the global level, was unanticipated.

eIF2α Phosphorylation Selectively Regulates Translation of Mitochondrial RPs

To investigate translation attenuation in more depth, we then focused on the RPs as a representative set of targets (Figures 3G and 3H). It is well established that cytosolic RPs are positively regulated by mTORC1 (Albert and Hall, 2015; Guan et al., 2014), but the regulation of mitochondrial RPs is unclear. Thus, we next examined if mTORC1 regulates translation of mitochondrial RPs. The translation of a subset of mitochondrial RPs was reported to be attenuated after a 12-h treatment with the dual mTORC1/C2 inhibitor PP242 (Morita et al., 2013). However, unlike their cytoplasmic counterparts, the mRNAs encoding mitochondrial RPs were found to be insensitive to an acute treatment with the selective mTORC1 inhibitor Torin1 in a dataset analyzed from a previous study (Figure 4A) (Thoreen et al., 2012), a finding confirmed here (Figure S6). This indicated that mitochondrial RPs are not directly regulated by mTORC1 during acute ER stress. Thus, we next asked whether they were regulated by eIF2α. In cells unable to phosphorylate eIF2α, which do not attenuate translation in response to stress (Scheuner et al., 2001) (Figure 4B), the translation downregulation of mitochondrial RPs by Tm was eliminated (Figure 4C). This establishes that the translation downregulation of mitochondrial RPs is under the control of eIF2α, an unanticipated finding. These proteins provide an ideal group to elucidate the features of the translation attenuation triggered by eIF2α.

5′ Untranslated mRNA Regions (UTRs) Are Sufficient for Translation Attenuation

We next asked how this selective translational attenuation was achieved. Knowing that the presence of upstream open reading frames (uORF) confers resistance to translation attenuation to ~10 mRNAs following eIF2α phosphorylation (Andreev et al., 2015; Hinnebusch, 2014; Young and Wek, 2016), we examined uORF content at the global level in the translationally regulated mRNAs, exploiting a systematic analyses of uORF in vertebrates (Johnstone et al., 2016). We found that at a global level, downregulated mRNAs had a lower uORF content (~0.5 in average) than mRNAs resistant to translational attenuation (Figure 5A). This raised the possibility that the 5′ UTRs of the translationally downregulated mRNAs might be important for this regulation. To test this hypothesis, we took advantage of a reporter system developed by Andreev and colleagues to study the mRNAs resistant to translation attenuation mediated by eIF2α phosphorylation (Andreev et al., 2015). We generated reporters containing 5′ UTR regions of the most translationally downregulated mRNAs identified in this study upstream of a firefly luciferase coding region. Capped and polyadenylated reporter mRNAs were prepared and transfected in mouse embryonic fibroblast (MEF) cells. We found that the reporters with the 5′ UTR regions of *Atp5d*, *Rps15*, *Rps15a*, *Rps18*, *Mrps28*, *Mrps33*, *Rps24*, and *Rpl35* were translationally downregulated 2 h after Tm treatment (Figure 5B), unlike the reporter with the 5′ UTR of ATF4, which was resistant to eIF2α phosphorylation (Figure 5B), as expected (Andreev et al., 2015). While the absence of uORFs in the 5′ UTR was a prevalent feature of the downregulated mRNAs at a global level (Figure 5A), the presence of one or two uORFs was insufficient to abolish translation attenuation of Rps24 and Rpl35 (Figure 5B). The same observation was made in NIH 3T3 cells, another mouse cell line (Figure S7). Importantly, translation attenuation of the responsive reporters was abolished in MEFs incompetent for eIF2α phosphorylation (Figure 5C). This establishes that translation attenuation was recapitulated on reporters harboring the 5′ UTR regions of translationally downregulated mRNAs.

Translationally Downregulated mRNAs Encode for Particularly Stable Proteins

Mutations in genes encoding RPs are deleterious, with dysfunction of both cytosolic and mitochondrial RPs being associated with severe human disorders (De Silva et al., 2015; Mills and Green, 2017). A preferential decrease in translation of such essential proteins seems surprising, because it comes with a

Figure 3. Global Changes in the Translatome upon Acute ER Stress

(A) Scatterplot of fold changes (Tm 2 h versus 0 h) as determined by anota2seq using total or polysome-associated mRNAs. Transcripts with changes in translational efficiency are shown in orange (up) or purple (down).

(B) Same as (A), but for Tm 4 h versus 0 h.

(C) *Rps6* and *Mrps16* mRNA abundances across the polysomal fractions at 2 and 4 h following Tm injection quantified by qPCR.

(D) Same as (C), with *Asns* and *Pah* mRNAs at 2 h and *Nedd4* and *Cul4b* mRNAs at 4 h following Tm injection.

In (C) and (D), representative results of three independent experiments are shown; data are mean ± SEM (n = 3).

(E) Boxplot of translation activities as determined by anota2seq of mRNAs downregulated at 2 h following Tm. The group of proteins synthetized at the ER were defined by Jan et al. (2014).

(F) Same as (E) but at 4 h following Tm.

(G) Boxplot of translation activities as determined by anota2seq of all mRNAs downregulated (Down), all mRNAs downregulated excluding the mRNAs encoding for the RPs (Down w/o RP), cytosolic RP mRNAs (CytoRP), and mitochondrial RP mRNAs (MitoRP), respectively, at 2 h following Tm.

(H) Same as (G), but 4 h following Tm.

(I) Density plot of translational activities of mRNAs downregulated at 2 h following Tm and represented at 0, 2, and 4 h following Tm.

(J) Same as (I), but with translational activities of downregulated mRNAs at 2 h represented at 0 and 10 h following Tm.

***$p < 0.001$, as determined by an unpaired Wilcoxon test. See also Figures S2–S5.

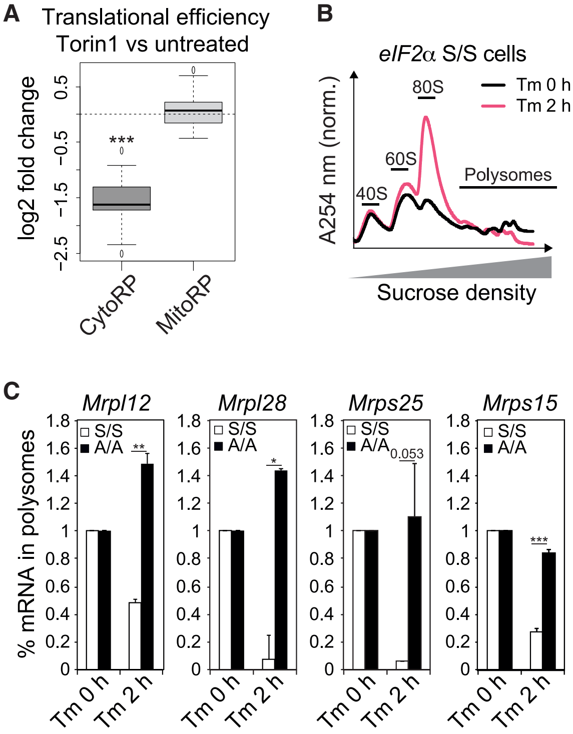

Figure 4. eIF2α Phosphorylation Attenuates Translation of Mitochondrial RPs

(A) Boxplots of Torin1-induced changes in translation efficiency for all mRNAs encoding CytoRPs and MitoRPs analyzed from Thoreen et al. (2012). ***$p < 0.001$, as determined by an unpaired Wilcoxon test.

(B) Representative polysome profiles from *eIF2α* S/S and *eIF2α* A/A MEFs at 0 and 2 h following treatment with 2.5 μg/mL Tm.

(C) *Mrpl12*, *Mrpl28*, *Mrps25*, and *Mrps15* mRNA abundance from heavy polysomal fractions (more than three ribosomes) shown in (B) assessed by qPCR. *$p < 0.05$, **$p < 0.01$, and ***$p < 0.001$, as determined by an unpaired Student t test. Representative results of at least three independent experiments are shown. Data are mean ± SEM (n = 3). See also Figure S6.

risk of compromising cell viability. Knowing that phosphorylation of eIF2α is an evolutionarily conserved response to stress, we reasoned that mechanisms ought to exist to safeguard against this threat. To shed light on this problem, we next aimed at measuring the cost resulting from decreasing translation of these essential proteins. We measured translation in the mitochondria, because the mitochondrial translation machinery is entirely distinct from the cytosolic one (O'Brien, 2003) and because we have shown that mitochondrial RPs are pure eIF2α targets, unlike cytosolic ones, which are known mTORC1 targets (Figures 4A, 4C, and S6). We used a sensitive method to specifically measure mitochondrial translation by metabolic labeling under conditions where cytoplasmic translation was inhibited with emetine (Grollman, 1968). As expected, chloramphenicol, a known inhibitor of mitochondrial translation (Bulkley et al., 2010; Münch and Harper, 2016), robustly decreased mitochondrial translation (Figure 6A). In contrast, Tm did not affect mitochondrial translation (Figure 6A). This shows that the decreased translation of mitochondrial RPs revealed here by the anota2seq analysis (Figures 3G, 3H, and S5) occurs without measurable functional consequences.

To elucidate the molecular basis of this conundrum, we next measured the steady-state abundance of a subset of RPs during translation attenuation. We observed that the translational downregulation of RPL4, RPS6, MRPL44, and MRPL28 occurred without measurable changes in their abundance (Figure 6B). This result is consistent with the finding that mitochondrial translation appeared unaffected by Tm treatment (Figure 6A). This reveals that translation attenuation did not result in a significant depletion of the targeted proteins. We do not rule out that the abundance of the translationally downregulated proteins decreases upon stress, but the decrease is below the detection levels of the analyses. This implies that translation attenuation occurs at minimal costs.

To elucidate the mechanism accounting for this puzzling observation, we measured the stability of the RPs. Cytosolic RPs are thought to have long half-lives (Nikolov et al., 1987). Confirming this notion, cytosolic RPs were stable over a 10 h block of protein synthesis using cycloheximide (Figure 6C). Likewise, mitochondrial RPs were also stable over 10 h (Figure 6C). In contrast, the levels of the short-lived protein POMP (Heink et al., 2005) were markedly decreased after 2-h cycloheximide treatment (Figure 6C), confirming the efficacy of the cycloheximide treatment.

These results suggested that translation attenuation following eIF2α phosphorylation might have evolved to preferentially target stable proteins, in order to minimize the cost of this stress response. To test this model, we compared how protein stability partitioned in our different datasets on a global scale using experimentally determined half-lives of mouse proteins (Sandoval et al., 2013). Remarkably, while protein half-lives in the two groups span from below 10 h to 100 h, we found that the proteins whose translation decreased after 2 h of Tm treatment were, as a group, significantly more stable than those resistant to translational attenuation (Figures 6D and 6E). These results were confirmed when using a different dataset of protein half-lives (Schwanhäusser et al., 2013) (Figure S8). Within the downregulated group, both cytoplasmic and mitochondrial RPs had the longest half-lives (Figure 6E). The findings that the translationally downregulated group encode for more stable proteins than the resistant ones remained significant upon exclusion of the RPs (Figure 6F), confirming that long half-life is a feature common to the entire group of downregulated proteins.

Importantly, we also found that the downregulated mRNAs encoded for more stable proteins when analyzing a ribosome profiling dataset where Tm was used to induce ER stress in HEK293T cells (Sidrauski et al., 2015) (Figures S9A and S9B). This feature was also observed upon analysis of a ribosome

CellPress
OPEN ACCESS

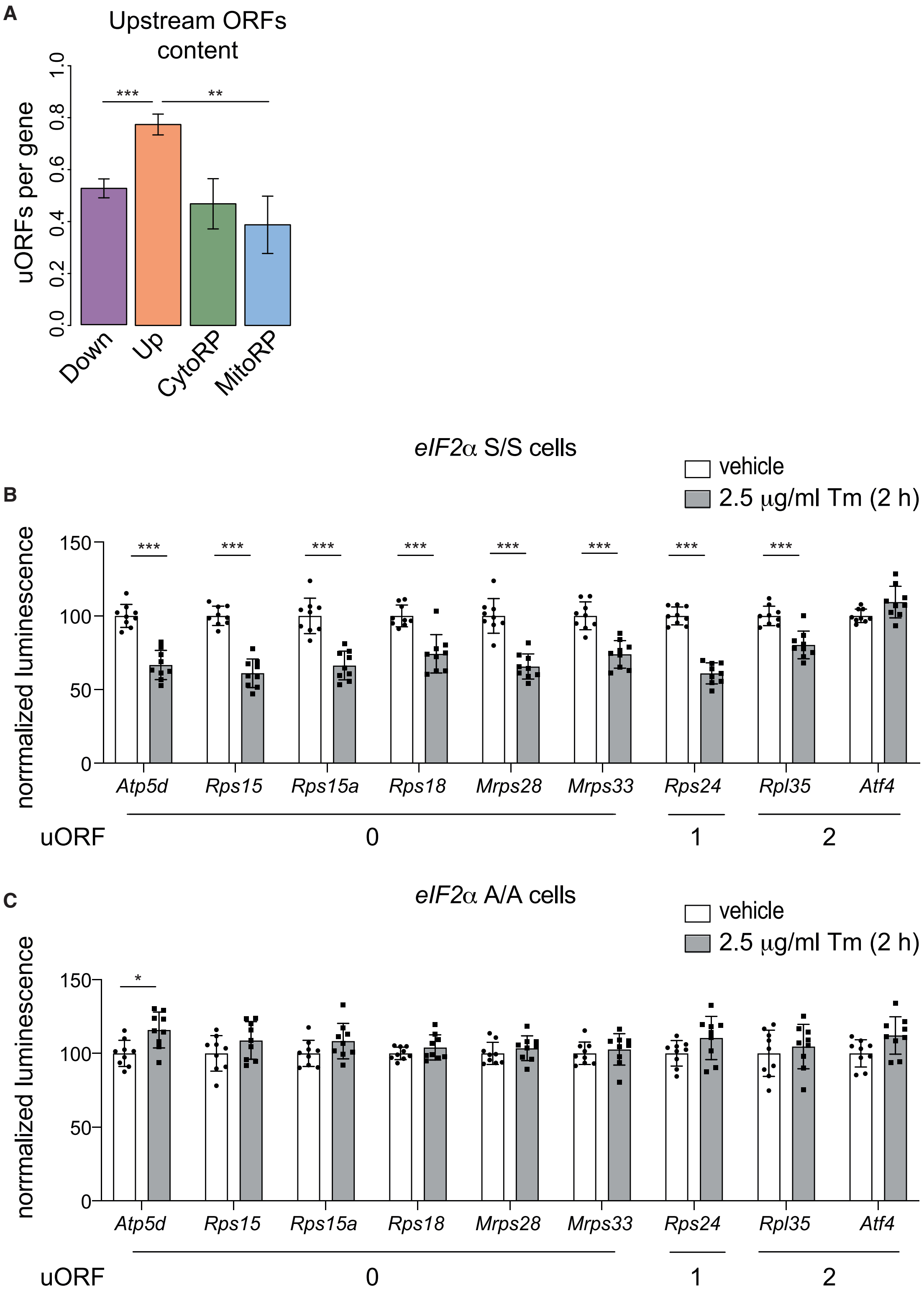

Figure 5. 5′ UTRs Are Sufficient to Provide Translation Attenuation following eIF2α Phosphorylation

(A) Upstream open reading frame (uORF) content in translationally down- or upregulated (Down or Up) mRNAs, and all 79 CytoRP mRNAs and all 82 MitoRP mRNAs 2 h following Tm injection. uORF content in mRNAs was analyzed from Johnstone et al. (2016). **$p < 0.01$ and ***$p < 0.001$ as determine by an unpaired Wilcoxon test.

(legend continued on next page)

profiling data studying early response to arsenite (Andreev et al., 2015), another eIF2α stressor (Figures S9C and S9D). The long half-lives of proteins encoded by downregulated mRNAs remained significant in both studies even after exclusion of the ribosomal RPs from the analysis (Figure S9). This shows that at a global level and in multiple studies, with different stressors and different methods used to measure translation, the translation attenuation resulting from eIF2α phosphorylation preferentially targets mRNAs encoding for long-lived proteins. It could be interesting to compare this response at the single mRNA level in side-by-side experiments in the future.

DISCUSSION

Here, we show that the translation attenuation following eIF2α phosphorylation is not uniform across all coding transcripts but preferentially affects mRNAs encoding long-lived proteins. As a result, the benefit of translation attenuation occurs at minimal cost, because it is mitigated by the stability of the targeted proteins. This reveals an additional component of the mechanism and evolutionary design of adaptation to ER stress.

The eIF2α-mediated attenuation of protein synthesis is a vital response to ER stress. Indeed, cells lacking eIF2α kinase PERK die when exposed to ER stress (Harding et al., 2000b). Likewise, cells harboring an unphosphorylable allele of eIF2α also fail to attenuate translation and die when exposed to stress (Scheuner et al., 2001). Abrogation of this adaptive translation attenuation in mice, through either the lack of eIF2α kinase PERK or a phosphorylable eIF2α, causes neonatal mortality (Harding et al., 2001; Scheuner et al., 2001). Similarly, in humans, loss of PERK function causes the rare, autosomal-recessive Wolcott-Rallison syndrome with neonatal to infancy onset and death before adulthood (Delépine et al., 2000). Thus, there is a large and consistent body of evidence establishing the vital importance of eIF2α-mediated translation attenuation for cell and organismal survival. However, there is equally abundant evidence establishing that general translation inhibitors such as cycloheximide are extremely toxic in cells and *in vivo* (Godchaux et al., 1967; McKeehan and Hardesty, 1969).

One vital feature of stress responses is their transient nature (Lamech and Haynes, 2015). The deleterious consequences of a persistent attenuation of translation are safeguarded by the two eIF2α phosphatases. Supporting this, the lack of the two eIF2α phosphatases is incompatible with life in mice (Harding et al., 2009), and a hypomorphic allele of R15B was also found deleterious in humans (Abdulkarim et al., 2015; Kernohan et al., 2015). We were surprised to find here that the mRNAs translationally downregulated 4 h following Tm treatment remained downregulated after translation recovery was manifest in the polysomal fractions (Figures 3I and 1E–1H). This implies that the recovery of translation observed at the global level, concomitant to eIF2α dephosphorylation, is actually not a recovery per se but most probably contributed by the increased translation of transcriptionally induced mRNAs. Thus, the translation attenuation of target mRNAs persists for longer than previously anticipated.

One of the proposed benefits of the translation attenuation response to ER stress was a reduction of the load of ER clients (Harding et al., 2002). Curiously, we find no enrichment for proteins synthesized at the ER in the pool of eIF2α-downregulated targets (Figures 3E and 3F). In light of the proposed ER-specific output of PERK-induced translational attenuation, this result may seem surprising. However, when considered in a broader context, it is a plausible result. Indeed, eIF2α phosphorylation is a common signaling event induced by one of four kinases (GCN2, PERK, HRI, and PKR), only one of which (PERK) is ER localized. The four ISR kinases evolved from a single eukaryotic ancestor, GCN2. In yeast, the cytosolic GCN2 responds to amino acid limitation by phosphorylating eIF2α (Wek, 2018), thereby reducing translation as well as the consumption of amino acids. PERK evolved with metazoan, and although PERK is an ER-transmembrane protein, its effector function, phosphorylation of eIF2α, is cytosolic. From this evolutionary perspective, the downregulation of translation following eIF2α phosphorylation evolved with no specificity for targets synthetized at the ER. Another mechanism has been proposed to reduce the flux of proteins in the ER during stress that consists of releasing mRNAs from the site of translation at the ER (Reid et al., 2014).

With a global analysis of translation attenuation following eIF2α phosphorylation *in vivo*, we found here that this stress response does not result in a uniform decrease of translation of all transcripts but preferentially targets some. Importantly, we observed that the transient stress-induced decreased translation of these targets occurs without measurably decreasing their abundance. Thus, the benefits of translation attenuation are achieved by reducing the production, but not the abundance, of the targeted proteins. Because protein synthesis is one of the most energy-consuming process, an immediate benefit to translation attenuation might be to spare energy. In addition, translation attenuation spares the consumption of amino acids, as well as protein quality control components (chaperones, proteasome) that are otherwise engaged with newly synthetized proteins. Attenuating translation of highly expressed proteins enables a significant effect on global translational activity with relatively small effects at the level of individual targets, an efficient way to reduce the cost of this stress response.

Our analysis shows that protein stability is a common property of the targets of translation attenuation following eIF2α phosphorylation, a feature that applies at a global level. Importantly, the same features were observed in different studies with different eIF2α stressors. This reveals that the translation attenuation mediated by eIF2α preferentially targets stable proteins, a design probably evolutionarily selected to minimize the cost of this adaptive response.

(B) Firefly luciferase activity in wild-type MEF cells (*eIF2α* S/S) transfected with reporter mRNAs containing the 5′ UTRs of indicated mRNAs and treated with either vehicle or 2.5 μg/mL Tm. Firefly luciferase activity was normalized against a transfection control (see STAR Methods for details) and vehicle-treated cells. Statistical analysis was carried out in Prism 8 using two-way ANOVA.
(C) Same as in (B), but in MEFs cells harboring an unphosphorylable eIF2α (*eIF2α* A/A).
Data in (B) and (C) are mean ± SEM (n = 9; three technical replicates in three independent experiments). See also Figure S7.

CellPress
OPEN ACCESS

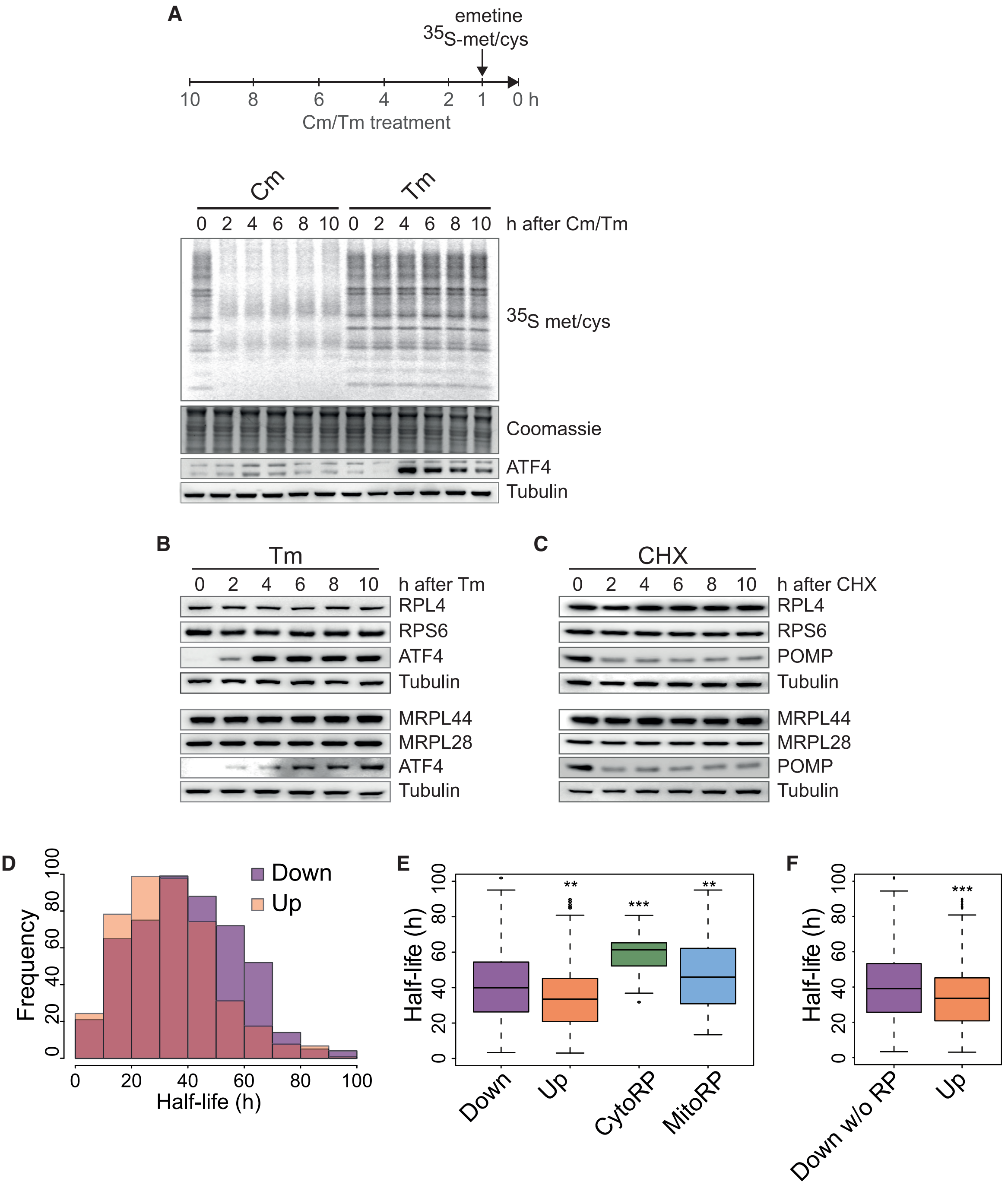

Figure 6. Translationally Downregulated Proteins following eIF2α Phosphorylation Are Long-Lived

(A) Experimental design (top panel) to measure mitochondrial translation. ^{35}S methionine/cysteine incorporation into newly translated mitochondrial proteins (encoded by mtDNA) following chloramphenicol (Cm; 50 μg/mL) or Tm (2.5 μg/mL) treatment of HEK293T cells for the indicated times. Cytoplasmic translation was inhibited using emetine (100 μg/mL). ATF4 was used as a positive control for the Tm treatment. Representative results of at least three independent experiments are shown.

(B) Representative immunoblots showing the abundance of RPL4, RPS6, MRPL44, MRPL28, ATF4, and tubulin proteins at the indicated times after treatment of HeLa cells with 2.5 μg/mL Tm. ATF4 was used as a positive control for the Tm treatment.

(C) Same as (B), but following 20 μg/mL cycloheximide (CHX) treatment. POMP (a short-lived protein) was used as a control for the CHX treatment.

(D) Histogram showing the distribution of protein half-lives encoded by translationally down- or upregulated mRNAs (Down or Up) at 2 h following Tm injection.

(legend continued on next page)

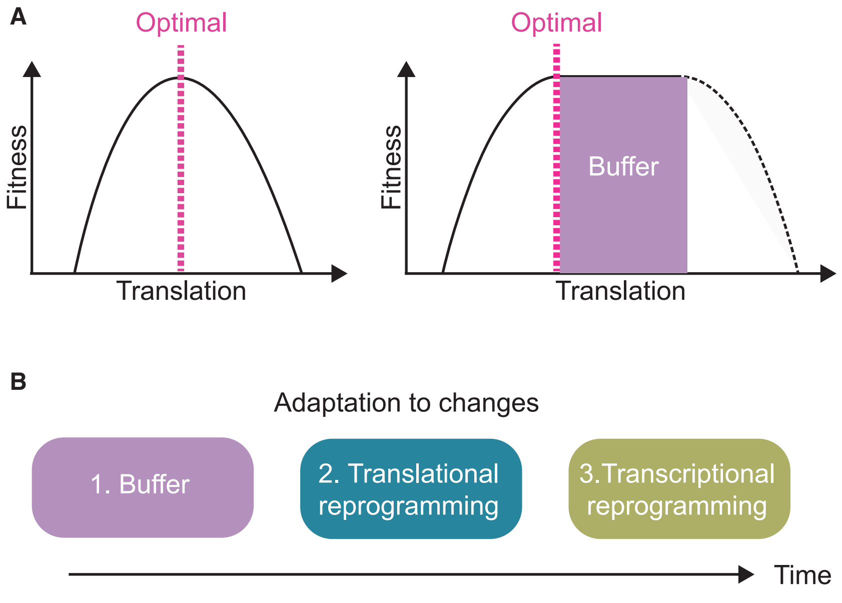

Figure 7. A Spare Capacity as an Immediately Available Buffer to Adapt to Changes

(A) A proposed model for rules governing protein translation rates. We propose that the rate of protein translation is set at higher levels than what is minimally required in order to provide a buffering capacity.

(B) The ER stress response is a three-tier system. A spare buffering capacity in cellular components provide immediate resources to buffer changes before translational and transcriptional reprogramming.

Protein abundance is thought to have been evolutionarily optimized to avoid wasting energy in producing excessive amounts (Dekel and Alon, 2005). Yet, a narrow match between an organism and its environment would restrict fitness to a limited set of conditions and compromise survival upon sudden changes. The observed decreased translation of some proteins at no measurable cost implies that these proteins may be synthesized above minimal requirements in unstressed conditions to provide a buffer for abrupt changes in conditions (Figure 7A). It is not known at present whether the proteins translated above the minimally required amount are degraded or if they are present above minimal requirements. It is, however, important to note other examples supporting the notion that protein abundance might be set above the minimal amount required to sustain viability. A recent theoretical study in bacteria also provides evidence for the existence of a spare capacity in ribosomes (Korem Kohanim et al., 2018). This comes at the cost of slower growth but enables rapid growth after an upshift in nutrients (Korem Kohanim et al., 2018). A similar principle of a spare capacity applies to the proteasome, with only ~20% of proteasome complexes found engaged in substrate processing under nonstressed conditions (Asano et al., 2015). This indicates that there might be as much as 80% of proteasomes free under basal conditions (Asano et al., 2015). If we consider the cost of producing and assembling a large multisubunit complex such as the proteasome (Rousseau and Bertolotti, 2018), there ought to have been selective evolutionary pressure to produce five times more than what is used under optimal conditions.

The following theory emerges from these examples and the present study: there might be a selective advantage to set protein translation at higher levels than what is minimally required in order to provide an immediately available buffer to rapidly adapt to abrupt changes in conditions (Figure 6A). Thus, in addition to the well-established translational and transcriptional changes that evolved to protect against ER stress, we suggest here an additional component to this adaptive response. We propose that the ER stress response is a three-tier system. The first tier is a spare buffering capacity in critical cellular components to provide an immediately available pool of resources to buffer changes instantaneously. The second tier is translation attenuation, a rapid, but not immediate, adaptive mechanism (Figures 1E–1G). The third mechanism, taking longer to implement, consists in transcriptional changes (Figure 1D and 7B).

This model leads to the following theory: protein translation and abundance of cellular components have been evolutionarily tuned by a tradeoff between optimality and adaptability. The abundance of proteins ought to be determined by not only the cost-benefit principle but also the selective pressure to keep a spare capacity to enable rapid adaptation to changes. This theory might be relevant to a broad range of biological systems.

STAR★METHODS

Detailed methods are provided in the online version of this paper and include the following:

- KEY RESOURCES TABLE
- RESOURCE AVAILABILITY
  - Lead Contact
  - Materials Availability
  - Data and Code Availability
- EXPERIMENTAL MODEL AND SUBJECT DETAILS
  - Animal Procedure

(E) Boxplot of half-lives of proteins encoded by the translationally down- or upregulated mRNAs (Down or Up), all 79 CytoRPs, or all 82 MitoRPs at 2 h following Tm injection.
(F) Same as (E), but with exclusion of the RPs (Down w/o RPs).
In (E) and (F), protein half-lives were analyzed from Sandoval et al. (2013). **p < 0.01 and ***p < 0.001, as determined by an unpaired Wilcoxon test. See also Figures S8 and S9.

- Cell lines
- METHOD DETAILS
  - Polysome purification
  - RNA isolation
  - Quantitative RT-PCR
  - RNA-seq
  - RNA-seq analysis
  - Gene ontology enrichment analysis
  - Computational analysis of already published datasets
  - Upstream ORF analysis
  - Luciferase reporter assay
  - Immunoblots
  - Mitochondrial translation
  - Assessment of translation rates
- QUANTIFICATION AND STATISTICAL ANALYSIS

SUPPLEMENTAL INFORMATION

Supplemental Information can be found online at https://doi.org/10.1016/j.celrep.2020.108154.

ACKNOWLEDGMENTS

We are grateful to the de Bono lab for assistance with bioinformatics, R. Kaufman for sharing *eIF2α* S/S and *eIF2α* A/A, and D. Andreev for the human ATF4 and R15B reporters. We are also grateful to C. Oertlin, J. Lorent, and O. Larsson for sharing a beta version of the anota2seq software, as well as O. Larsson for helpful suggestions. We thank the sequencing facility at the CRUK institute, especially J. Hadfield; the J. Chin and V. Ramakrishnan labs for support; I. S. Fernández for advice on polysome fractionation; and P. Freire-Pritchett, T.J. Stevens, M. Babu, M. Goedert, U. Alon, M. Schuldiner, G. Borner, and the members of the Bertolotti lab for discussions and/or critical comments on the manuscript. This work was supported by the Medical Research Council (UK) grant MC_U105185860 and the European Research Council (ERC) under the European Union's Seventh Framework Programme (FP7/2007- 2013)/ERC grant 309516 and a Wellcome Trust Principal Investigator Award (206367/Z/17/Z). K. Schneider was supported by the Swiss National Science Foundation, and A. Weber was funded by the Deutsche Forschungsgemeinschaft (DFG [German Research Foundation] grant 421713657).

AUTHOR CONTRIBUTIONS

K.S. designed, performed, and analyzed the study; prepared the figures; and helped with the manuscript. G.M.N. and J.L.W. performed the computational analyses with help from J.M. L.M.L. performed the analyses of mitochondrial translation. A.W. performed the eIF2α blots and the 5′ UTR analysis with constructs cloned by M.D. A.B. supervised the study and wrote the manuscript.

DECLARATION OF INTERESTS

The authors declare no competing interests.

Received: March 18, 2020
Revised: May 25, 2020
Accepted: August 25, 2020
Published: September 15, 2020

REFERENCES

Abdulkarim, B., Nicolino, M., Igoillo-Esteve, M., Daures, M., Romero, S., Philippi, A., Senée, V., Lopes, M., Cunha, D.A., Harding, H.P., et al. (2015). A missense mutation in PPP1R15B causes a syndrome including diabetes, short stature, and microcephaly. Diabetes *64*, 3951–3962.

Albert, V., and Hall, M.N. (2015). mTOR signaling in cellular and organismal energetics. Curr. Opin. Cell Biol. *33*, 55–66.

Andreev, D.E., O'Connor, P.B., Fahey, C., Kenny, E.M., Terenin, I.M., Dmitriev, S.E., Cormican, P., Morris, D.W., Shatsky, I.N., and Baranov, P.V. (2015). Translation of 5′ leaders is pervasive in genes resistant to eIF2 repression. eLife *4*, e03971.

Asano, S., Fukuda, Y., Beck, F., Aufderheide, A., Förster, F., Danev, R., and Baumeister, W. (2015). Proteasomes. A molecular census of 26S proteasomes in intact neurons. Science *347*, 439–442.

Bazzini, A.A., Johnstone, T.G., Christiano, R., Mackowiak, S.D., Obermayer, B., Fleming, E.S., Vejnar, C.E., Lee, M.T., Rajewsky, N., Walther, T.C., and Giraldez, A.J. (2014). Identification of small ORFs in vertebrates using ribosome footprinting and evolutionary conservation. EMBO J. *33*, 981–993.

Bertolotti, A. (2018). The split protein phosphatase system. Biochem. J. *475*, 3707–3723.

Brostrom, C.O., and Brostrom, M.A. (1998). Regulation of translational initiation during cellular responses to stress. Prog. Nucleic Acid Res. Mol. Biol. *58*, 79–125.

Brostrom, M.A., Prostko, C.R., Gmitter, D., and Brostrom, C.O. (1995). Independent signaling of grp78 gene transcription and phosphorylation of eukaryotic initiator factor 2 alpha by the stressed endoplasmic reticulum. J. Biol. Chem. *270*, 4127–4132.

Brostrom, C.O., Prostko, C.R., Kaufman, R.J., and Brostrom, M.A. (1996). Inhibition of translational initiation by activators of the glucose-regulated stress protein and heat shock protein stress response systems. Role of the interferon-inducible double-stranded RNA-activated eukaryotic initiation factor 2alpha kinase. J. Biol. Chem. *271*, 24995–25002.

Bulkley, D., Innis, C.A., Blaha, G., and Steitz, T.A. (2010). Revisiting the structures of several antibiotics bound to the bacterial ribosome. Proc. Natl. Acad. Sci. USA *107*, 17158–17163.

Buttgereit, F., and Brand, M.D. (1995). A hierarchy of ATP-consuming processes in mammalian cells. Biochem. J. *312*, 163–167.

Carrara, M., Sigurdardottir, A., and Bertolotti, A. (2017). Decoding the selectivity of eIF2α holophosphatases and PPP1R15A inhibitors. Nat. Struct. Mol. Biol. *24*, 708–716.

De Silva, D., Tu, Y.T., Amunts, A., Fontanesi, F., and Barrientos, A. (2015). Mitochondrial ribosome assembly in health and disease. Cell Cycle *14*, 2226–2250.

Dekel, E., and Alon, U. (2005). Optimality and evolutionary tuning of the expression level of a protein. Nature *436*, 588–592.

Delépine, M., Nicolino, M., Barrett, T., Golamaully, M., Lathrop, G.M., and Julier, C. (2000). EIF2AK3, encoding translation initiation factor 2-alpha kinase 3, is mutated in patients with Wolcott-Rallison syndrome. Nat. Genet. *25*, 406–409.

Dever, T.E., Feng, L., Wek, R.C., Cigan, A.M., Donahue, T.F., and Hinnebusch, A.G. (1992). Phosphorylation of initiation factor 2 alpha by protein kinase GCN2 mediates gene-specific translational control of GCN4 in yeast. Cell *68*, 585–596.

Godchaux, W., 3rd, Adamson, S.D., and Herbert, E. (1967). Effects of cycloheximide on polyribosome function in reticulocytes. J. Mol. Biol. *27*, 57–72.

Grollman, A.P. (1968). Inhibitors of protein biosynthesis. V. Effects of emetine on protein and nucleic acid biosynthesis in HeLa cells. J. Biol. Chem. *243*, 4089–4094.

Guan, B.J., Krokowski, D., Majumder, M., Schmotzer, C.L., Kimball, S.R., Merrick, W.C., Koromilas, A.E., and Hatzoglou, M. (2014). Translational control during endoplasmic reticulum stress beyond phosphorylation of the translation initiation factor eIF2α. J. Biol. Chem. *289*, 12593–12611.

Guan, B.J., van Hoef, V., Jobava, R., Elroy-Stein, O., Valasek, L.S., Cargnello, M., Gao, X.H., Krokowski, D., Merrick, W.C., Kimball, S.R., et al. (2017). A unique ISR program determines cellular responses to chronic stress. Mol. Cell *68*, 885–900.e6.

Harding, H.P., Novoa, I., Zhang, Y., Zeng, H., Wek, R., Schapira, M., and Ron, D. (2000a). Regulated translation initiation controls stress-induced gene expression in mammalian cells. Mol. Cell *6*, 1099–1108.

Harding, H.P., Zhang, Y., Bertolotti, A., Zeng, H., and Ron, D. (2000b). Perk is essential for translational regulation and cell survival during the unfolded protein response. Mol. Cell *5*, 897–904.

Harding, H.P., Zeng, H., Zhang, Y., Jungries, R., Chung, P., Plesken, H., Sabatini, D.D., and Ron, D. (2001). Diabetes mellitus and exocrine pancreatic dysfunction in perk-/- mice reveals a role for translational control in secretory cell survival. Mol. Cell *7*, 1153–1163.

Harding, H.P., Calfon, M., Urano, F., Novoa, I., and Ron, D. (2002). Transcriptional and translational control in the Mammalian unfolded protein response. Annu. Rev. Cell Dev. Biol. *18*, 575–599.

Harding, H.P., Zhang, Y., Scheuner, D., Chen, J.J., Kaufman, R.J., and Ron, D. (2009). Ppp1r15 gene knockout reveals an essential role for translation initiation factor 2 alpha (eIF2alpha) dephosphorylation in mammalian development. Proc. Natl. Acad. Sci. USA *106*, 1832–1837.

Heink, S., Ludwig, D., Kloetzel, P.M., and Krüger, E. (2005). IFN-gamma-induced immune adaptation of the proteasome system is an accelerated and transient response. Proc. Natl. Acad. Sci. USA *102*, 9241–9246.

Hinnebusch, A.G. (2014). The scanning mechanism of eukaryotic translation initiation. Annu. Rev. Biochem. *83*, 779–812.

Hinnebusch, A.G., Ivanov, I.P., and Sonenberg, N. (2016). Translational control by 5′-untranslated regions of eukaryotic mRNAs. Science *352*, 1413–1416.

Ingolia, N.T., Brar, G.A., Rouskin, S., McGeachy, A.M., and Weissman, J.S. (2013). Genome-wide annotation and quantitation of translation by ribosome profiling. Curr. Protoc. Mol. Biol. *Chapter 4*, Unit 4.18–4.18.19.

Jan, C.H., Williams, C.C., and Weissman, J.S. (2014). Principles of ER cotranslational translocation revealed by proximity-specific ribosome profiling. Science *346*, 1257521.

Johnstone, T.G., Bazzini, A.A., and Giraldez, A.J. (2016). Upstream ORFs are prevalent translational repressors in vertebrates. EMBO J. *35*, 706–723.

Kaufman, R.J. (2004). Regulation of mRNA translation by protein folding in the endoplasmic reticulum. Trends Biochem. Sci. *29*, 152–158.

Kernohan, K.D., Tétreault, M., Liwak-Muir, U., Geraghty, M.T., Qin, W., Venkateswaran, S., Davila, J., Holcik, M., Majewski, J., Richer, J., and Boycott, K.M.; Care4Rare Canada Consortium (2015). Homozygous mutation in the eukaryotic translation initiation factor 2alpha phosphatase gene, PPP1R15B, is associated with severe microcephaly, short stature and intellectual disability. Hum. Mol. Genet. *24*, 6293–6300.

Kilkenny, C., Browne, W.J., Cuthill, I.C., Emerson, M., and Altman, D.G. (2012). Improving bioscience research reporting: the ARRIVE guidelines for reporting animal research. PLoS Biol. *8*, e1000412.

Kim, D., Pertea, G., Trapnell, C., Pimentel, H., Kelley, R., and Salzberg, S.L. (2013). TopHat2: accurate alignment of transcriptomes in the presence of insertions, deletions and gene fusions. Genome Biol. *14*, R36.

Korem Kohanim, Y., Levi, D., Jona, G., Towbin, B.D., Bren, A., and Alon, U. (2018). A bacterial growth law out of steady state. Cell Rep. *23*, 2891–2900.

Krishnamoorthy, T., Pavitt, G.D., Zhang, F., Dever, T.E., and Hinnebusch, A.G. (2001). Tight binding of the phosphorylated alpha subunit of initiation factor 2 (eIF2alpha) to the regulatory subunits of guanine nucleotide exchange factor eIF2B is required for inhibition of translation initiation. Mol. Cell. Biol. *21*, 5018–5030.

Krzyzosiak, A., Sigurdardottir, A., Luh, L., Carrara, M., Das, I., Schneider, K., and Bertolotti, A. (2018). Target-based discovery of an inhibitor of the regulatory phosphatase PPP1R15B. Cell *174*, 1216–1228.e19.

Lamech, L.T., and Haynes, C.M. (2015). The unpredictability of prolonged activation of stress response pathways. J. Cell Biol. *209*, 781–787.

Larsson, O., Sonenberg, N., and Nadon, R. (2011). anota: analysis of differential translation in genome-wide studies. Bioinformatics *27*, 1440–1441.

Larsson, O., Morita, M., Topisirovic, I., Alain, T., Blouin, M.J., Pollak, M., and Sonenberg, N. (2012). Distinct perturbation of the translatome by the antidiabetic drug metformin. Proc. Natl. Acad. Sci. USA *109*, 8977–8982.

Love, M.I., Huber, W., and Anders, S. (2014). Moderated estimation of fold change and dispersion for RNA-seq data with DESeq2. Genome Biol. *15*, 550.

Ly, T., Endo, A., Brenes, A., Gierlinski, M., Afzal, V., Pawellek, A., and Lamond, A.I. (2018). Proteome-wide analysis of protein abundance and turnover remodelling during oncogenic transformation of human breast epithelial cells. Wellcome Open Res. *3*, 51.

McKeehan, W., and Hardesty, B. (1969). The mechanism of cycloheximide inhibition of protein synthesis in rabbit reticulocytes. Biochem. Biophys. Res. Commun. *36*, 625–630.

Merico, D., Isserlin, R., Stueker, O., Emili, A., and Bader, G.D. (2010). Enrichment map: a network-based method for gene-set enrichment visualization and interpretation. PLoS ONE *5*, e13984.

Mills, E.W., and Green, R. (2017). Ribosomopathies: there's strength in numbers. Science *358*, eaan2755.

Morita, M., Gravel, S.P., Chénard, V., Sikström, K., Zheng, L., Alain, T., Gandin, V., Avizonis, D., Arguello, M., Zakaria, C., et al. (2013). mTORC1 controls mitochondrial activity and biogenesis through 4E-BP-dependent translational regulation. Cell Metab. *18*, 698–711.

Münch, C., and Harper, J.W. (2016). Mitochondrial unfolded protein response controls matrix pre-RNA processing and translation. Nature *534*, 710–713.

Nikolov, E.N., Dineva, B.B., Dabeva, M.D., and Nikolov, T.K. (1987). Turnover of ribosomal proteins in regenerating rat liver after partial hepatectomy. Int. J. Biochem. *19*, 159–163.

Novoa, I., Zhang, Y., Zeng, H., Jungreis, R., Harding, H.P., and Ron, D. (2003). Stress-induced gene expression requires programmed recovery from translational repression. EMBO J. *22*, 1180–1187.

O'Brien, T.W. (2003). Properties of human mitochondrial ribosomes. IUBMB Life *55*, 505–513.

Oertlin, C., Lorent, J., Murie, C., Furic, L., Topisirovic, I., and Larsson, O. (2019). Generally applicable transcriptome-wide analysis of translation using anota2seq. Nucleic Acids Res. *47*, e70.

Oyadomari, S., Harding, H.P., Zhang, Y., Oyadomari, M., and Ron, D. (2008). Dephosphorylation of translation initiation factor 2alpha enhances glucose tolerance and attenuates hepatosteatosis in mice. Cell Metab. *7*, 520–532.

Pilla, E., Schneider, K., and Bertolotti, A. (2017). Coping with protein quality control failure. Annu. Rev. Cell Dev. Biol. *33*, 439–465.

Reid, D.W., Chen, Q., Tay, A.S.L., Shenolikar, S., and Nicchitta, C.V. (2014). The unfolded protein response triggers selective mRNA release from the endoplasmic reticulum. Cell *158*, 1362–1374.

Rolfe, D.F., and Brown, G.C. (1997). Cellular energy utilization and molecular origin of standard metabolic rate in mammals. Physiol. Rev. *77*, 731–758.

Ron, D., and Harding, H.P. (2012). Protein-folding homeostasis in the endoplasmic reticulum and nutritional regulation. Cold Spring Harb. Perspect. Biol. *4*, a013177.

Rousseau, A., and Bertolotti, A. (2018). Regulation of proteasome assembly and activity in health and disease. Nat. Rev. Mol. Cell Biol. *19*, 697–712.

Sandoval, P.C., Slentz, D.H., Pisitkun, T., Saeed, F., Hoffert, J.D., and Knepper, M.A. (2013). Proteome-wide measurement of protein half-lives and translation rates in vasopressin-sensitive collecting duct cells. J. Am. Soc. Nephrol. *24*, 1793–1805.

Scheuner, D., Song, B., McEwen, E., Liu, C., Laybutt, R., Gillespie, P., Saunders, T., Bonner-Weir, S., and Kaufman, R.J. (2001). Translational control is required for the unfolded protein response and in vivo glucose homeostasis. Mol. Cell *7*, 1165–1176.

Schwanhäusser, B., Busse, D., Li, N., Dittmar, G., Schuchhardt, J., Wolf, J., Chen, W., and Selbach, M. (2013). Corrigendum: Global quantification of mammalian gene expression control. Nature *495*, 126–127.

Sidrauski, C., McGeachy, A.M., Ingolia, N.T., and Walter, P. (2015). The small molecule ISRIB reverses the effects of eIF2α phosphorylation on translation and stress granule assembly. eLife *4*, e05033.

Sonenberg, N., and Hinnebusch, A.G. (2009). Regulation of translation initiation in eukaryotes: mechanisms and biological targets. Cell *136*, 731–745.

Stoneley, M., Paulin, F.E., Le Quesne, J.P., Chappell, S.A., and Willis, A.E. (1998). C-Myc 5′ untranslated region contains an internal ribosome entry segment. Oncogene *16*, 423–428.

Thoreen, C.C., Chantranupong, L., Keys, H.R., Wang, T., Gray, N.S., and Sabatini, D.M. (2012). A unifying model for mTORC1-mediated regulation of mRNA translation. Nature *485*, 109–113.

Trapnell, C., Roberts, A., Goff, L., Pertea, G., Kim, D., Kelley, D.R., Pimentel, H., Salzberg, S.L., Rinn, J.L., and Pachter, L. (2012). Differential gene and transcript expression analysis of RNA-seq experiments with TopHat and Cufflinks. Nat. Protoc. *7*, 562–578.

Wang, M., and Kaufman, R.J. (2016). Protein misfolding in the endoplasmic reticulum as a conduit to human disease. Nature *529*, 326–335.

Wek, R.C. (2018). Role of eIF2α kinases in translational control and adaptation to cellular stress. Cold Spring Harb. Perspect. Biol. *10*, a032870.

Wiseman, R.L., Haynes, C.M., and Ron, D. (2010). SnapShot: the unfolded protein response. Cell *140*, 590–590.e2.

Wong, W.L., Brostrom, M.A., Kuznetsov, G., Gmitter-Yellen, D., and Brostrom, C.O. (1993). Inhibition of protein synthesis and early protein processing by thapsigargin in cultured cells. Biochem. J. *289*, 71–79.

Yamamoto, K., Takahara, K., Oyadomari, S., Okada, T., Sato, T., Harada, A., and Mori, K. (2010). Induction of liver steatosis and lipid droplet formation in ATF6alpha-knockout mice burdened with pharmacological endoplasmic reticulum stress. Mol. Biol. Cell *21*, 2975–2986.

Young, S.K., and Wek, R.C. (2016). Upstream open reading frames differentially regulate gene-specific translation in the integrated stress response. J. Biol. Chem. *291*, 16927–16935.

STAR★METHODS

KEY RESOURCES TABLE

REAGENT or RESOURCE	SOURCE	IDENTIFIER
Antibodies		
Mouse monoclonal CHOP	Thermo Fisher Scientific	MA1-250, RRID:AB_2292611
Mouse monoclonal p-eIF2α	Thermo Fisher Scientific	44-728G, RRID:AB_2533736
Mouse monoclonal tubulin	Sigma-Aldrich	T5168, RRID:AB_477579
Rabbit polyclonal MRPL44	Proteintech	16394-1-AP, RRID:AB_2146062
Rabbit monoclonal MRPL28	Abcam	ab126719, RRID:AB_11133067
Rabbit polyclonal RPL4	Proteintech	11302-1-AP, RRID:AB_2181909
Rabbit monoclonal RPS6	Cell Signaling	2217S, RRID:AB_331355
Rabbit monoclonal HSPC014	Abcam	ab170865
Rabbit monoclonal phospho-S6 ribosomal protein	Cell Signaling	4856S, RRID:AB_2181037
Mouse monoclonal ATF4	Santa Cruz Biotechnology	sc-390063, RRID:AB_2810998
Chemicals, Peptides, and Recombinant Proteins		
Tunicamycin	Sigma-Aldrich	T7765-5MG
Cycloheximide	Sigma-Aldrich	Cat#C7698
SYBR green	ThermoFisher Scientific	Cat#44-729-08
Luciferase RNA	Promega	L4561
SuperScript II	ThermoFisher Scientific	18064014
SuperScript IV	ThermoFisher Scientific	18091050
Critical Commercial Assays		
RNeasy Mini Kit	QIAGEN	Cat#74104
iScript™ cDNA Synthesis Kit	Biorad	1708890
TruSeq Stranded mRNA Library Prep Kit	Illumina	20020595
RiboMAX™ Large Scale RNA Production Kit	Promega	P1300
Dual-Luciferase Reporter Assay System	Promega	E1910
Cells		
HEK293T. Sex undetermined.	ATCC	CRL-3216
MEF cells: *eIF2α* S/S. Sex undetermined.	Scheuner et al., 2001	N/A
MEF cells: *eIF2α* A/A. Sex undetermined.	Scheuner et al., 2001	N/A
NIH 3T3 cells	ATCC	LGC Standards Teddington, UK
Experimental Models: Organisms/Strains		
C57BL/6J	The Jackson Laboratory	Cat#000664
Oligonucleotides		
Oligonucleotides	Sigma-Aldrich	Table S2
Software and Algorithms		
GraphPad Prism 7	GraphPad Software	https://www.graphpad.com/scientific-software/prism/
R studio	R Studio	https://rstudio.com/products/rstudio/
Cytoscape	Cytoscape	https://cytoscape.org/
PlotDigitizer	PlotDigitizer	https://sourceforge.net/projects/plotdigitizer/
Biocomp gradient station software	Biocomp	N/A
TopHat v2.0.13	Kim et al., 2013	N/A

(Continued on next page)

Continued

REAGENT or RESOURCE	SOURCE	IDENTIFIER
Cufflinks suite v2.2.1	Trapnell et al., 2012	N/A
Cuffdiff	Trapnell et al., 2012	N/A
anota2seq v1.0.0	Oertlin et al., 2019	N/A
gplots (v3.0.1)	R Studio	N/A
DAVID	DAVID bioinformatics Resources 6.8	https://david.ncifcrf.gov/home.jsp
Data deposition		
Sequencing data	NCBI Gene Expression Omnibus	GSE156335

RESOURCE AVAILABILITY

Lead Contact

Further information and requests for reagents should be directed to and will be fulfilled by the Lead Contact, Anne Bertolotti (aberto@mrc-lmb.cam.ac.uk).

Materials Availability

This study did not generate new unique reagents.

Data and Code Availability

The accession number for the sequencing data reported in this paper is GEO: GSE156335.

EXPERIMENTAL MODEL AND SUBJECT DETAILS

Animal Procedure

Ethical statement on mouse studies

All animal care and procedures were performed in compliance with the regulation on the use of Animals in Research (UK Animals Scientific Procedures Act of 1986 and the EU Directive 2010/63/EU) under the project license number 70/7956 and with approval from the LMB Animal Welfare and Ethical Review committee.

Housing and husbandry of experimental animals

All experimental animals were housed and cared according to the Home Office Code of Practice for the Housing and Care of Animals used in Scientific Procedures. In this study, the ARRIVE guidelines have been followed (Kilkenny et al., 2012). Experimental mice were kept in specific pathogen free ventilated cages (Tecniplast GM500, Techniplast) on Lignocel FS14 spruce bedding (IPS, Ltd.) and Enviro-Dri nesting material (LBS) at 19-23°C with 12 hours light dark cycle with light from 7.00 am to 7.00 pm. Animals were fed with Dietex CRM pellets (Special Diet Services) with free access on food and water.

For the tunicamycin (Tm) experiment, 15 5-week-old C57BL/6 (The Jackson Laboratory) males were used. Males were weaned at 3-4 weeks of ages and 2 to 3 animals were caged together. Mice were injected intraperitoneally (IP) with 0.1 mg/kg Tm (Sigma-Aldrich) at 8 am. The day before injection, animals were weighted, and average weight was used to calculate the volume of Tm injected. Mice were culled at 10 am (2 hours), noon (4 hours), 2 pm (6 hours) and 6 pm (10 hours) hours by cervical dislocation, respectively, and livers were collected. The control mice (0 hours) were culled at 9 am.

Cell lines

HeLa, HEK293T and NIH 3T3 cells were cultured in Dulbecco's Modified Eagle's Media (DMEM, ThermoFisher Scientific) supplemented with penicillin, streptomycin, and glutamine (Pen Step Glu, ThermoFisher Scientific) and 10% fetal bovine serum (FBS, ThermoFisher Scientific). *eIF2*α S/S and *eIF2*α A/A MEFs cells were cultures in DMEM supplemented with Pen Strep Glu, 55 μM β-mercaptoethanol, 1 X non-essential amino acids (ThermoFisher Scientific) and 10% FBS. All mammalian cell lines were grown in a humidified incubator with 5% CO_2 at 37°C.

METHOD DETAILS

Polysome purification

From liver

Livers were collected form C57BL/6 animals at the indicated times and directly flash frozen in liquid nitrogen. For polysome analysis, 0.25 g of liver were homogenized in 500 μL of lysis buffer containing 20 mM HEPES pH 7.6, 250 mM NaCl, 10 mM $MgCl_2$, 10 mM DTT

(Sigma-Aldrich), 20 μg/ml cycloheximide (Sigma-Aldrich), 2.5 μl/ml RNase inhibitor (RNasin ® Ribonuclease Inhibitors, Promega), protease inhibitor cocktails (Roche Life Science) and phosphatase inhibitor tablets (PhosSTOP, Roche Life Science). The homogenates were centrifuged 10 minutes at 9,500 g and 4°C to remove the cellular debris and 1 mg/ml heparin (Sigma-Aldrich), 0.5% Na deoxycholate, and 0.5% Triton X-100 were added to the supernatant. Cells were lysed by passing extracts 10 times through 27-gauge syringe needles. 50 mg of lysates were deposited in a 36 mL 7%–47% sucrose gradient containing 20 mM HEPES pH 7.6, 100 mM KCl, 5 mM $MgCl_2$ and 1 mM DTT. Gradients were centrifuged for 4 hours 30 minutes at 140,000 g and 4°C and divided in 1.5 mL fractions using a peristaltic pump. Optical density of the fractions was continuously measured at 260 nm to establish polysomal profiles. Polysomal profiles were digitalized using the PlotDigitizer software (https://sourceforge.net/projects/plotdigitizer/).

From cells

Cells were seeded in 15 cm dishes (1×10^5 cells/ml) and cultured overnight (1 dish of HeLa cells; 4 dishes of *eIF2α* S/S or A/A cells per condition). When indicated, cells were treated with 250 nM of Torin1 (Tocris Bioscience), DMSO or 2.5 μg/ml Tm for 2 hours, prior to polysome analysis. Cells were then incubated 5 minutes with 100 μg/ml of cycloheximide (CHX, Sigma-Aldrich), washed twice with ice-cold PBS containing 100 μg/ml of cycloheximide (PBS + CHX) and harvested in 10 mL of PBS + CHX. Cells were then spun for 5 minutes at 200 g and 4°C and resuspended in 425 μL of hypotonic buffer (5 mM Tris-HCl pH 7.5, 2.5 mM $MgCl_2$, 1.5 mM KCl, 1 x protease inhibitor cocktail EDTA free (Roche Life Science), 2.5 μL of 20 μg/ml CHX, 1 μL of 1M DTT, 100 U RNase inhibitor (RNasin® Ribonuclease Inhibitors, Promega)). After vortexing the samples for 5 s, 25 μL of 10% Triton X-100 and 25 μL of 10% sodium deoxycholate were added. The samples were then vortexed again for 5 s and centrifuged 7 minutes at 16,000 g and 4°C. 10% of the lysate was kept for determination of cytosolic steady-state mRNA levels and 430 μL were loaded in a 5%–50% sucrose gradient. The gradient was centrifuged at 36,000 g at 4°C for 2 hours and sampled using the Biocomp gradient station (Biocomp) with constant monitoring of optical density at 254 nm.

RNA isolation

From liver

Total RNAs were isolated from 0.01 g of liver using the RNeasy Mini Kit (QIAGEN) according to the manufacturer's instruction. For the isolation of the polysomal RNAs, the 'heavy' polysomal fractions (more than 3 ribosomes) were pooled and RNAs were precipitated in three volumes of 100% ethanol overnight at −20°C. The following day, RNAs were extracted using the RNeasy Mini Kit (QIAGEN) according to the manufacturer's instructions. To allow quantification of RNAs from polysomal fractions, 200 ng of Luciferase RNA was spiked in to every fraction prior to precipitation when indicated (Luciferase control RNA, Promega).

From cells

Heavy polysomal fractions as well as input mRNAs were precipitated overnight in three volumes of 100% ethanol and mRNAs were isolated the following morning using the RNeasy Mini Kit (QIAGEN) according to the manufacturer's instructions.

Quantitative RT-PCR

cDNAs were synthesized using the SuperscriptIV reverse transcriptase (ThermoFisher Scientific) according to the manufacturer's instructions. Transcript abundance was determined by quantitative PCR (qPCR) using SYBR Green PCR mix (ThermoFisher Scientific) in a Corbett Rotor Gene 6000 instrument (QIAGEN). Relative mRNA levels were calculated on the basis of 2CT and normalized to Cyclophilin B, Luciferase mRNA and/or total mRNA levels as indicated. The primers used for qPCRs analyses are listed in the Table S2.

RNA-seq

2 μg and 0.5 μg of total and polysomal RNAs, respectively, were used to prepare sequencing libraries using the TruSeq Stranded mRNA Library Prep Kit (Illumina) according to the manufacturer's instructions. All total and polysomal libraries were multiplexed into a single pool which was sequenced across 6 lanes, all on the same flow cell, on an Illumina HiSeq 2500. On average, each library generated approximately 17 million single-end 50 bp reads.

RNA-seq analysis

Transcript quantification and differential expression

TopHat v2.0.13 (Kim et al., 2013) was used (–no-coverage-search,–library-type fr-firststrand) to align reads to a transcriptome index built from the mouse reference genome GRCm38. Transcript abundances and differentially expressed mRNAs were determined by the Cufflinks suite v2.2.1 (Trapnell et al., 2012) (–library-type fr-firststrand,–frag-bias-correct,–multi-read-correct). Differentially expressed mRNAs were identified, via Cuffdiff (Trapnell et al., 2012), as those with a q-value < 0.05 and a fold change > = 2.

Translational efficiency analysis using anota2seq

Raw fragment counts for total and polysomal individual replicates were used as input to the anota2seq (v1.0.0) R package (Oertlin et al., 2019). Counts were normalized using the "TMM-log2" method via the anota2seqDataSetFromMatrix function. The resulting anota2seqDataSet object was then provided as input to the anota2seqRun wrapper function which was run using default parameters. mRNAs with significant changes in translational efficiency across conditions were determined using default thresholds within anota2seq. One of

these thresholds includes the maximum *p-value* (maxPAdj), adjusted for multiple testing via Benjamini-Hochberg correction, which was set to 0.15, as in previous anota analyses (Larsson et al., 2012; Oertlin et al., 2019). Also, the Random Variance Model was applied (useRVM = TRUE).

Cytosolic versus polysome-associated mRNA fold change plots were generated using the anota2seqPlotFC function. The heatmap.2 function belonging to the gplots (v3.0.1) R package was used to produce the translational efficiency heatmaps.

Gene ontology enrichment analysis

Sets of mRNAs of interest were analyzed using the gene ontology online tool DAVID (http://david.abcc.ncifcrf.gov/home.jsp). In order to cluster the related gene ontology categories, we use the enrichment map function in Cytoscape as in Merico et al. (2010). The input for the enrichment maps were the GOTERM_BP_FAT from DAVID, with the default Benjamini correction. The node size corresponds to the number of genes present under a given GO category. The bigger the node is, the more genes are falling under a giver GO category. The connectors between the nodes represent the overlap in genes between two GO categories. The broader the connectors are, the more genes are shared between two categories. Clusters of functionally related GO terms were automatically assigned by the software and highlighted.

Computational analysis of already published datasets

In Figures 3E and 3F, the proteins synthesized at the ER were defined based on a proximity-specific ribosome profiling dataset from Jan et al. (2014). ER proteins and others were mapped to our anota2seq data, and compared by nonparametric Wilcoxon test.

In Figure 4A, data were accessed from Thoreen et al. (2012). mRNAs with a 1.5 fold decrease in translational efficiency upon Torin-1 treatment were deemed translationally downregulated. This gene list was analyzed to assess mTOR-dependent and independent translational effects on mRNAs encoding ribosomal proteins.

In Figures 6D–6F, data were accessed from Sandoval et al. (2013), emanating from vehicle-treated mouse collecting duct cells (https://hpcwebapps.cit.nih.gov/ESBL/Database/ProteinHalfLives/index.html). Proteins with a robustly measured half-life (present in 3 or more samples) were identified. The results from this analysis were compared to our anota2seq analysis.

In Figure S8, data were accessed from Table S3 of Schwanhäusser et al. (2013), and protein half-lives, measured in mouse fibroblasts.

In Figures S9*A* and *S9B*, data from untreated and tunicamycin treated HEK293T cells (Sidrauski et al., 2015) were downloaded from the Gene Expression Omnibus (GSE65778). Expression levels were analyzed using DEseq2 (Love et al., 2014). mRNAs that underwent a significant up- or downregulation at the total translational level ($p < 0.05$) were mapped to half-life data generated in human cells (Ly et al., 2018), for genes where half-life data were available. Data were compared by nonparametric Wilcoxon test.

In Figures S9C and *S9D*, data generated in HEK293T cells were downloaded from Andreev et al. (2015) and the 300 most downregulated and the 300 least downregulated mRNAs, identified by ribosome profiling, were taken for downstream analysis. mRNAs for which half-life information was available were mapped to half-life data generated in human cells (Ly et al., 2018) and compared by nonparametric Wilcoxon test.

Upstream ORF analysis

Per-gene 5′UTR ORF frequencies, made available in a mouse-specific dataset (Johnstone et al., 2016), were contrasted for selected gene lists. These frequencies specifically included upstream ORFs (uORFs) and overlapping ORFs (oORFs), as annotated by the earlier study (Bazzini et al., 2014).

Luciferase reporter assay

Plasmid constructs

5′UTRs were amplified from cDNA generated with the iScript cDNA synthesis kit (Biorad) using total RNA isolated from mouse brain as template (for primer sequences see Table S2). The DNA fragments were cloned into pGL3 (Stoneley et al., 1998) using the restriction sites SpeI and NcoI (NEB), except for the 5′UTR of *Mrps28* cloned using EcoRI and NcoI and the one of *Atf4* using SpeI and EcoRI. Very short 5′UTRs were inserted into pGL3 directly using annealed forward and reverse primers (*Rps15*, *Rps15a* and *Rps18*).

mRNA preparation

mRNA preparation was performed as described by Andreev et al. (2015). Briefly, DNA templates for RNA production were obtained by PCR of pGL3 constructs (containing the 5’UTR of interest) using an universal forward primer binding upstream of the SpeI site and carrying the T7 promoter (CGCCGTAATACGACTCACTATAGGGAGCTTATCGATACCGTCG) and an universal reverse primer containing a stretch of 50 nucleotides of thymidine providing a poly-A tail to the mRNA (50TAACTTGTTTATTGCAGCTTATAATGG). PCR products were purified and used as templates for *in vitro* RNA transcription using the T7 RNA polymerase (RiboMAX^{TM} Large Scale RNA Production Kit, Promega). For m7G-capping of the RNA constructs, 3′-O-Me-m7GpppG (ARCA cap analog, NEB) was added to the reaction. Capped RNA constructs were purified with the RNeasy Mini Kit (QIAGEN). mRNAs integrity was examined by agarose gel electrophoresis.

Transfection of mRNA constructs and detection of bioluminescence

Cells were plated in 24-well plates the day before transfection in order to reach a final confluency between 70 to 80% (in triplicates for every condition). Cells were transfected with a mixture of 0.2 μg capped mRNA and a fluorescein-labeled dsRNA oligomer used as

transfection control (BLOCK-iT Fluorescence Oligo, ThermoFisher scientific, final concentration in well: 100 nM) using Lipofectamin 2000 (Invitrogen). One hour after transfection, cells were treated with either 2.5 μg/mL tunicamycin or vehicle for two hours. Cells were harvested using the Dual Luciferase Assay kit (Promega) and lysates transferred in 96-well plates. mRNAs luminescence was analyzed using a PHERAstar FSX plate reader (BMG Labtech) followed by fluorescence measurements of fluorescein-labeled dsRNA oligomer (tranfection control). The luminescence data for each well was normalized with the corresponding fluorescence signal from the transfection control. To be able to compare independent experiments, the average vehicle luminescence signal of each mRNA was set to 100. Experiments were performed in triplicate.

Immunoblots

From liver

10 mg of liver were lysed in 1 mL of RIPA buffer (1 mM EDTA, 50 mM Tris pH 7.4, 0.25% DOC, 0.1% SDS, 50 mM NaCl, 1% NP40, phosphatase inhibitor tablets (PhosSTOP, Roche Life Science), protease inhibitor cocktail (Roche Life Science) and 1 mM DTT). Extracts were centrifuged 30 minutes at 14,000 rpm and 4°C. Supernatants were collected and further diluted 1/20 in RIPA buffer. 50 μL of 4 times loading buffer (100 mM Tris pH 6.8, 20% glycerol, 4% SDS, 0.1% bromophenol blue and 200 mM DTT) was added to every 100 μL of extracts and 10 μL of extract were loaded on a 4%–12% Bolt Bis-Tris Plus gel (ThermoFisher Scientific).

From cells

HeLa cells or 293T cells were seeded in 12-well plate (2×10^5 cells/ml) 24 hours before each experiment. Immediately after the indicated treatments, cells were lysed using 120 μL of 2 x loading buffer (50 mM Tris pH 6.8, 10% glycerol, 2% SDS, 0.05% bromophenol blue and 100 mM DTT), boiled at 95°C for 5 minutes and sonicated. 10 μL of extract were loaded on a 4%–12% Bolt Bis-Tris Plus gel (ThermoFisher Scientific).

Immunoblots were visualized using the Biorad ChemiDoc^{TM} touch imaging system (Biorad).

The following antibodies were used: CHOP (Thermo Fisher Scientific, MA1-250, 1/500 dilution), p-eIF2α (ThermoFisher Scientific, 44-728G, 1/1000 dilution), tubulin (Sigma-Aldrich, T5168, 1/2000 dilution), MRPL44 (Proteintech, 16394-1-AP, 1/1000 dilution), MRPL28 (Abcam, ab126719, 1/1000 dilution), RPL4 (Proteintech, 11302-1-AP, 1/1000 dilution), RPS6 (Cell Signaling, 2217S, 1/1000 dilution), HSPC014 (Abcam, ab170865, 1/1000 dilution) phospho-S6 ribosomal protein (Cell Signaling, 4856S, 1/1000 dilution) and ATF4 (Santa Cruz Biotechnology, sc-390063, 1/1000 dilution).

Mitochondrial translation

Following the indicated treatments, 293T cells were incubated for 20 minutes in 90% methionine/cysteine-free DMEM (ThermoFisher Scientific) complemented with 5% dialyzed serum and 2 mM L-glutamine. Cells were then incubated for 1 hour in the same medium in the presence of 100 μg/ml emetine (Sigma-Aldrich) to inhibit cytoplasmic translation and 100 μCi/ml ^{35}S-labeled methionine/cysteine (EasyTag, PerkinElmer). Lysates were resolved on a 12% Bolt Bis-Tris Plus gel (ThermoFisher Scientific) and analyzed by autoradiography.

Assessment of translation rates

To assess translation rates, ^{35}S labeling of cells was performed as previously described (Krzyzosiak et al., 2018). Briefly, HeLa cells were plated ($1x10^5$ cells/ well) in 12-well plates the day prior to treatment. Cells were treated with 2.5 μg/mL Tunicamycin (Tm), incubated at 37°C for the indicated times and then labeled with 100 μCi/mL ^{35}S-methionine (Hartmann Analytic) for 10 min at 37°C. Labeled cells were washes twice with ice-cold PBS and harvested in Laemmli Buffer. Samples were boiled at 95°C for 10 min, sonicated, and resolved on 4-12% Bolt Bis-Tris Plus gels (ThermoFisher Scientific). After staining with InstantBlue (Expedeon), the gels were pre-treated with a solution of 20% ethanol, 7% acetic acid and 4% glycerol for 10 min and dried on a filter paper using a gel dryer. ^{35}S labeling was assessed using the Storage Phosphor Screen (GE Healthcare) and analyzed by phosphor-imaging using a Typhoon Imager Scanner (GE Healthcare). Lanes were quantified using ImageJ software.

QUANTIFICATION AND STATISTICAL ANALYSIS

Sample size for each experiment was estimated based on previous studies. For assessment of the effect of Tm *in vivo*, 3 animals per time points have been used (n = 3). For all cell experiments, a minimal of 3 biological replicates have been performed.

The statistical comparisons were carried out either in GraphPad Prism 7 or in R Studio using unpaired two-tailed Student t test or two-way ANOVA. For samples non-normally distributed, a Wilcoxon test was used . To assess whether two datasets were correlated, we used the Spearman correlation coefficient. All statistical methods and significant differences used are stated in the corresponding figures. Differences were considered as statistically significant at p values below 0.05.

The cut-offs used for all RNaseq experiments (including anota2seq analysis) are described in details in the corresponding method sections.

For the gene ontology analyses, the default Benjamini correction (from DAVID) was used.

All the data are represented as mean ± SEM except for polysomal traces, western blots and ^{35}S met/cyst labeling experiments where one representative result of at least three independent experiment is shown.

No samples, mouse or data points were excluded from the analyses.

Cell Reports, Volume *32*

Supplemental Information

Protein Stability Buffers the Cost of Translation Attenuation following eIF2α Phosphorylation

Kim Schneider, Geoffrey Michael Nelson, Joseph Luke Watson, Jörg Morf, Maximillian Dalglish, Laura Martina Luh, Annika Weber, and Anne Bertolotti

Supplemental Information

Table S1: Top 10 GO terms of translationally regulated mRNAs as determined by anota2seq, Related to Figure 1

Top 10 GO terms of relative translationally down- or upregulated mRNAs as determined by anota2seq at 2 and 4 hours following Tm injection.

Translationally downregulated mRNAs at 2 hours

Category	GO terms	count	%	p value	**adjusted p value**
GOTERM_BP_DIRECT	translation	136	8.2	1.90E-52	6.60E-49
GOTERM_BP_DIRECT	transport	232	14	2.50E-15	4.50E-12
GOTERM_BP_DIRECT	mitochondrial translation	21	1.3	1.00E-13	1.20E-10
GOTERM_BP_DIRECT	protein transport	99	6	2.40E-13	2.20E-10
GOTERM_BP_DIRECT	rRNA processing	36	2.2	1.10E-11	8.10E-09
GOTERM_BP_DIRECT	oxidation-reduction process	101	6.1	1.20E-10	7.30E-08
GOTERM_BP_DIRECT	glutathione metabolic process	21	1.3	1.50E-10	7.40E-08
GOTERM_BP_DIRECT	antigen processing and presentation of exogenous peptide antigen via MHC class I, TAP-dependent	16	1	9.10E-10	4.00E-07
GOTERM_BP_DIRECT	proton transport	21	1.3	4.90E-09	1.90E-06
GOTERM_BP_DIRECT	vesicle-mediated transport	43	2.6	1.20E-08	4.20E-06

Translationally downregulated mRNAs at 4 hours

Category	GO terms	count	%	p value	**adjusted p value**
GOTERM_BP_DIRECT	translation	105	10.	7.20E-50	1.80E-46
GOTERM_BP_DIRECT	transport	144	14.	4.90E-11	6.10E-08
GOTERM_BP_DIRECT	oxidation-reduction process	69	6.9	8.00E-10	6.60E-07
GOTERM_BP_DIRECT	proton transport	17	1.7	4.20E-09	2.60E-06
GOTERM_BP_DIRECT	ATP synthesis coupled proton	11	1.1	2.50E-08	1.30E-05
GOTERM_BP_DIRECT	ribosomal small subunit assembly	10	1	1.50E-07	6.20E-05
GOTERM_BP_DIRECT	cytoplasmic translation	12	1.2	1.90E-07	6.60E-05
GOTERM_BP_DIRECT	mitochondrial translation	12	1.2	3.60E-07	1.10E-04
GOTERM_BP_DIRECT	rRNA processing	20	2	4.50E-06	1.20E-03
GOTERM_BP_DIRECT	ATP metabolic process	11	1.1	9.20E-06	2.30E-03

Translationally upregulated mRNAs at 2 hours

Category	GO terms	count	%	p value	**adjusted p value**
GOTERM_BP_DIRECT	mRNA processing	95	5.1	3.50E-25	1.60E-21
GOTERM_BP_DIRECT	metabolic process	116	6.2	6.20E-24	1.50E-20
GOTERM_BP_DIRECT	RNA splicing	70	3.8	2.40E-18	3.80E-15
GOTERM_BP_DIRECT	protein transport	110	5.9	1.00E-12	1.20E-09
GOTERM_BP_DIRECT	covalent chromatin modification	61	3.3	4.10E-11	3.80E-08
GOTERM_BP_DIRECT	transcription, DNA-templated	255	13.	6.70E-11	5.30E-08
GOTERM_BP_DIRECT	flavonoid biosynthetic process	15	0.8	1.80E-10	1.20E-07

GOTERM_BP_DIRECT	flavonoid glucuronidation	15	0.8	1.80E-10	1.20E-07
GOTERM_BP_DIRECT	oxidation-reduction process	113	6.1	4.30E-10	2.50E-07
GOTERM_BP_DIRECT	DNA repair	65	3.5	1.40E-09	7.40E-07

Translationally upregulated mRNAs at 4 hours

Category	GO terms	count	%	p value	**adjusted p value**
GOTERM_BP_DIRECT	flavonoid glucuronidation	12	1.8	6.20E-12	1.40E-08
GOTERM_BP_DIRECT	flavonoid biosynthetic process	12	1.8	6.20E-12	1.40E-08
GOTERM_BP_DIRECT	xenobiotic glucuronidation	9	1.4	7.20E-12	8.20E-09
GOTERM_BP_DIRECT	metabolic process	38	5.7	1.50E-07	1.20E-04
GOTERM_BP_DIRECT	transcription, DNA-templated	93	14	6.90E-06	3.90E-03
GOTERM_BP_DIRECT	mRNA processing	25	3.8	7.20E-05	3.20E-02
GOTERM_BP_DIRECT	protein ubiquitination involved in ubiquitin-dependent protein catabolic process	15	2.3	7.30E-05	2.80E-02
GOTERM_BP_DIRECT	regulation of transcription, DNA-	103	15.	7.40E-05	2.40E-02
GOTERM_BP_DIRECT	response to nutrient	10	1.5	4.10E-04	1.10E-01
GOTERM_BP_DIRECT	protein ubiquitination	25	3.8	4.20E-04	1.00E-01

Table S2: Oligonucleotides used in this study

*MmPpp1r15a*_R: tctcaggtcctccttcctca	Sigma-Aldrich	N/A
*MmChop*_F: cctagcttggctgacagagg	Sigma-Aldrich	N/A
*MmChop*_R: ctgctccttctccttcatgc	Sigma-Aldrich	N/A
*MmBiP*_F: ctttgatcagcgggtcatgg	Sigma-Aldrich	N/A
*MmBiP*_R: agctcttcaaatttggcccg	Sigma-Aldrich	N/A
*MmGrp94*_F: agtcgggaagcaacagagaa	Sigma-Aldrich	N/A
*MmGrp94*_R: tctccatgttgccagaccat	Sigma-Aldrich	N/A
*MmNedd4*_F: atgtggatgctgggagttga	Sigma-Aldrich	N/A
*MmNedd4*_R: ctgcacgctggtaaggattc	Sigma-Aldrich	N/A
*MmRps6*_F: aaggtaagaagcccaggacc	Sigma-Aldrich	N/A
*MmRps6*_R: ctgttcctggcgcttttctt	Sigma-Aldrich	N/A
*MmCul4b*_F: cccttgtcttccttgcacac	Sigma-Aldrich	N/A
*MmCul4b*_R: aggaagcaaagcagagtcct	Sigma-Aldrich	N/A
*MmAsns*_F: aggaggcatcgagaaaacca	Sigma-Aldrich	N/A
*MmAsns*_R: aagaagggagtggtggagtg	Sigma-Aldrich	N/A
HsRpl4_F: tgggacgtttctgcatttgg	Sigma-Aldrich	N/A
HsRpl4_R: cgatggatcttcttgcgtgg	Sigma-Aldrich	N/A
HsMrpl12_F: ggaggcggtggaagaagata	Sigma-Aldrich	N/A
HsMrpl12_R: gactccaccagcttctttgc	Sigma-Aldrich	N/A
HsMrpl28_F: ggacctgtgctccaagtttg	Sigma-Aldrich	N/A
HsMrpl28_R: ttgaacaggggtacagggtc	Sigma-Aldrich	N/A
HsMrps15_F: ggccaacaagaaggagatgc	Sigma-Aldrich	N/A
HsMrps15_R: ttgtgggctttgtcctttcg	Sigma-Aldrich	N/A
HsMrps25_F: cagggaagaggaggaggaga	Sigma-Aldrich	N/A
HsMrps25_R: aacccttgggtgcagatcat	Sigma-Aldrich	N/A
MmMrpl12_F: tcctcctcctcttccctgag	Sigma-Aldrich	N/A
MmMrpl12_R: cctccaaggctgctttgatc	Sigma-Aldrich	N/A
MmMrpl28_F: ccccggtacactacaaacct	Sigma-Aldrich	N/A
MmMrpl28_R: tccacaccttcttcaccctc	Sigma-Aldrich	N/A
MmMrps15_F: caagcaggacgatgaaccac	Sigma-Aldrich	N/A
MmMrps15_R: attgcggatcctgacagtca	Sigma-Aldrich	N/A
MmMrps25_F: cccttcctgcggttctatct	Sigma-Aldrich	N/A

MmMrps25_R: ggccttctacttcgcacatg	Sigma-Aldrich	N/A
*Cyclophilin B*_F: ggagatggcacaggaggaa	Sigma-Aldrich	N/A
*Cyclophilin B*_R: gcccgtagtgcttcagctt	Sigma-Aldrich	N/A
*Luciferase*_F: aaaggatatcaggtggcccc	Sigma-Aldrich	N/A
*Luciferase*_R: ccacaaacacaactcctccg	Sigma-Aldrich	N/A
*Atp5d*_5UTR_F: gatacaactagtccttcggagaatcctgtgc	Sigma-Aldrich	N/A
*Atp5d*_5UTR_R: gatacaccatggggtggcggaaaagtcagtg	Sigma-Aldrich	N/A
*Atf4*_5UTR_F: gatacaactagtggctaggtgtcccaccc	Sigma-Aldrich	N/A
*Atf4*_5UTR_R: gatacagaattcgttgtggggctttgctggattcg	Sigma-Aldrich	N/A
*Mrps28*_5UTR_F: gatacagaattcaggcaagctttaaactaccggac	Sigma-Aldrich	N/A
*Mrps28*_5UTR_R: gatacaccatgggacgcctgcgcgacctc	Sigma-Aldrich	N/A
*Mrps33*_5UTR_F: gatacaactagttccgggttgtccgccgac	Sigma-Aldrich	N/A
*Mrps33*_5UTR_R: gatacaccatggttcttcaccagtagggaacaggattcc	Sigma-Aldrich	N/A
*Rps15a*_5UTR_F: gatacaactagtataattccgtgcgtttgcccggcccactctctttctgccatcttccctcgccgccacc	Sigma-Aldrich	N/A
*Rps15a*_5UTR_R: gatacaccatggggtggcggcgagggaagatggcagaaagagagtgggccgggcaaacgcacggaattat	Sigma-Aldrich	N/A
*Rps18*_5UTR_F: gatacaactagtgtcacttccgctctctcttccacaggaggcctacacgccgccgtctgtgccgccgcc	Sigma-Aldrich	N/A
*Rps18*_5UTR_R: gatacaccatggggcggcggcacagacggcggcgtgtaggcctcctgtggaagagagagcggaagtgac	Sigma-Aldrich	N/A
*Rpl35*_5UTR_F: ggactagtgaacagtggaagaagagaactc	Sigma-Aldrich	N/A
*Rpl35*_5UTR_F: catgccatggttcggcgttgttcacg	Sigma-Aldrich	N/A
*Rps24*_5UTR_F: ggactagtgctggggtccttcctt	Sigma-Aldrich	N/A
*Rps24*_5UTR_R: catgccatgggatggctacggcgcc	Sigma-Aldrich	N/A
*Rps15*_5UTR_F: gatacaactagtgtacttccttttccgagtaaccgccaag	Sigma-Aldrich	N/A
*Rps15*_5UTR_R: gatacaccatggcttggcggttactcggaaaaggaagtac	Sigma-Aldrich	N/A

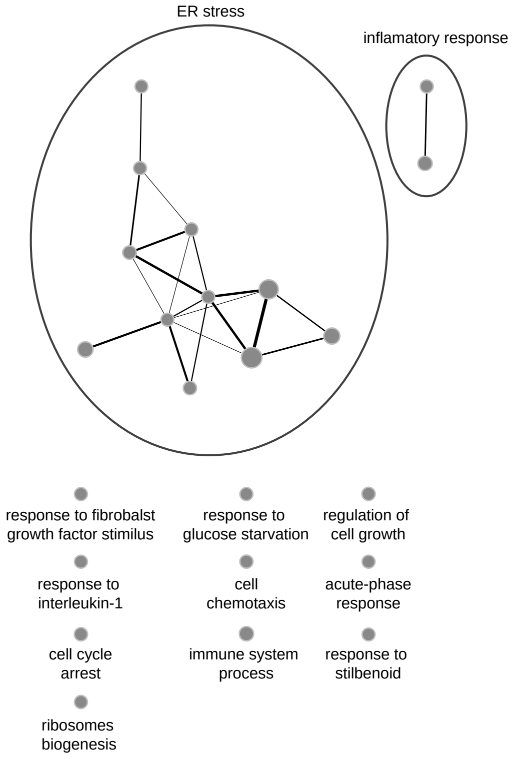

Figure S1. Gene ontology enrichment map of mRNAs transcriptionally upregulated after Tm, Related to Figure 1

Significant (P<0.001) gene ontology enrichment maps of the 900 mRNAs transcriptionally upregulated (2-fold cut-off) 4 hours after Tm injection.

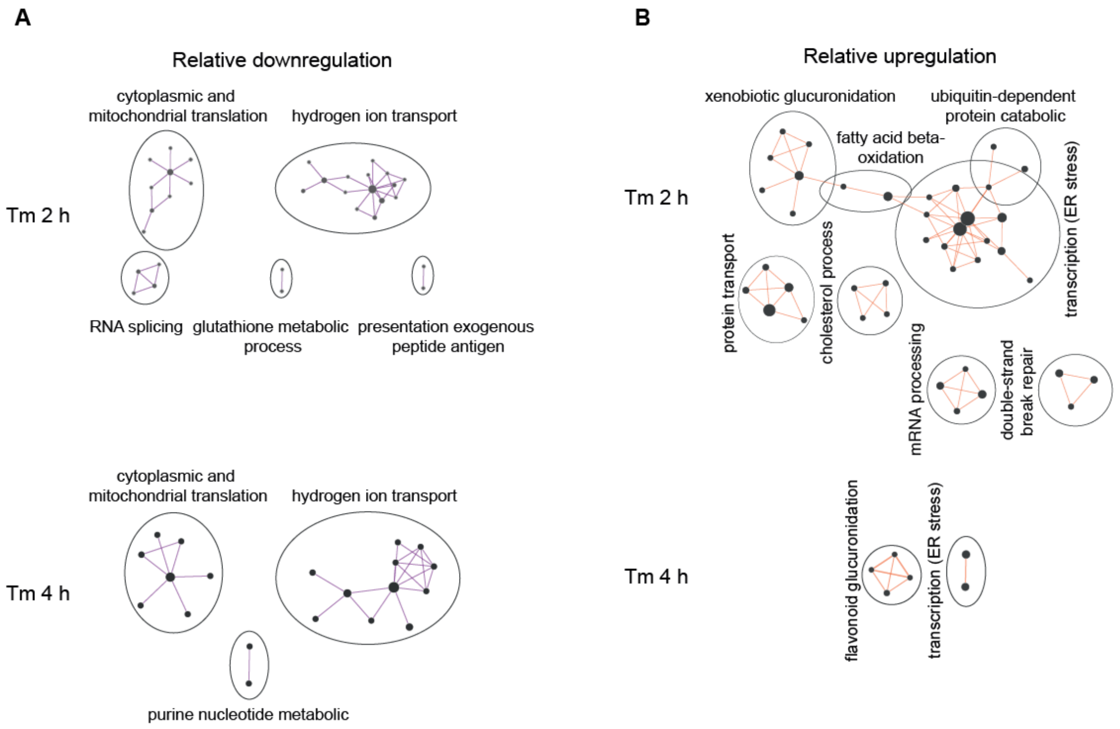

Figure S2. GO analysis of mRNA translationally affected following Tm, Related to Figure 3

(A) Significant (P<0.001) GO enrichment maps of translationally downregulated mRNAs as determined by anota2seq at 2 and 4 hours following Tm injection.

(B) Same as (A) for genes resistant to translational attenuation (relative upregulation).

A

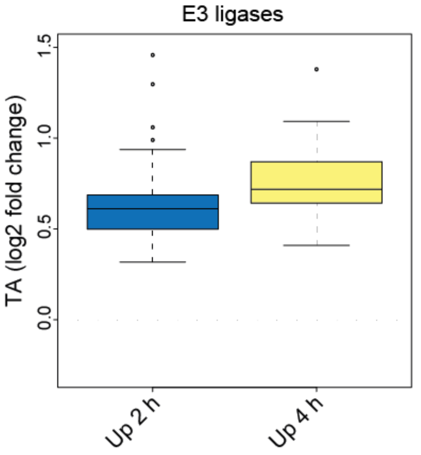

B

E3 ligases upregulated (relative upregulation) at 2 h

Klhl9	*Ubr2*	*Fbxo11*	*Brca1*
Klhl28	*Trip12*	*Usp36*	*G2E3*
Klhdc7A	*Bag6*	*Trim23*	*Usp34*
Cdc73	*Ddb1*	*Ubr3*	*Fbxo21*
Pja2	*Dzip3*	*Rnf111*	*Rnf123*
Klhl22	*Usp24*	*Fbxo10*	*Usp33*
Cand1	*Asb3*	*Cyld*	*Usp22*
Usp4	*Nfe2L2*	*Usp9X*	*Dcaf8*
Shprh	*Abtb1*	*Dcaf17*	*Ddb2*
Usp47	*Tnks*	*Sash1*	*Usp48*
Leo1	*Usp19*	*Pdzrn3*	
Ltn1	*Hectd3*	*Kbtbd4*	
Uvssa	*Pja1*	*Ctr9*	
Hltf	*Trim33*	*Cdc20*	
Med17	*Sqstm1*	*Ttc3*	

E3 ligases upregulated (relative upregulation) at 4 h

Klhl28	*Nedd4*
Crbn	*Usp47*
Socs4	*Ubr5*
Klhl24	*Arntl*
Vps4B	*Fbxo21*
Klhl22	*Fbxo30*
Cul4B	*Ube3A*
Rnf216	*Mib2*
Vps37A	*Asb3*
Klhdc7A	*Cbll1*
Rnf125	
Usp34	
Ubr7	
Cyld	
Lrrc41	

Figure S3. Relative translational regulation of E3 ligases following Tm, Related to Figure 3
(A) Boxplot showing the median translation efficiency (TA) as determined by anota2seq at 2 and 4 hours following Tm treatment.
(B) List of E3 ligases translationally upregulated (relative upregulation) at 2 and 4 hours following Tm, respectively.

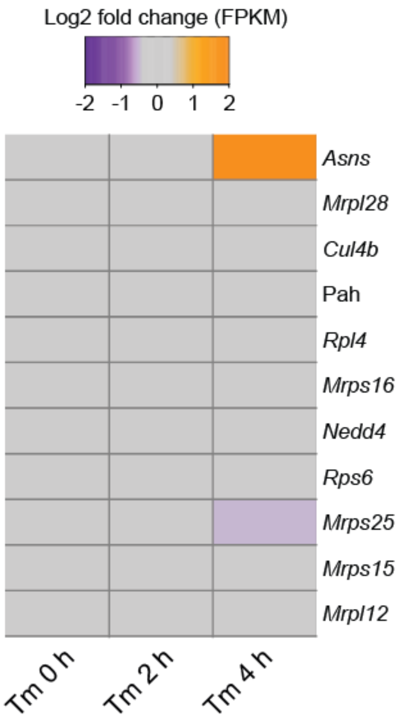

Figure S4. mRNAs abundance of selected transcripts, Related to Figure 3
Log2 FPKM values for indicated genes at 0, 2 and 4 hours following Tm.

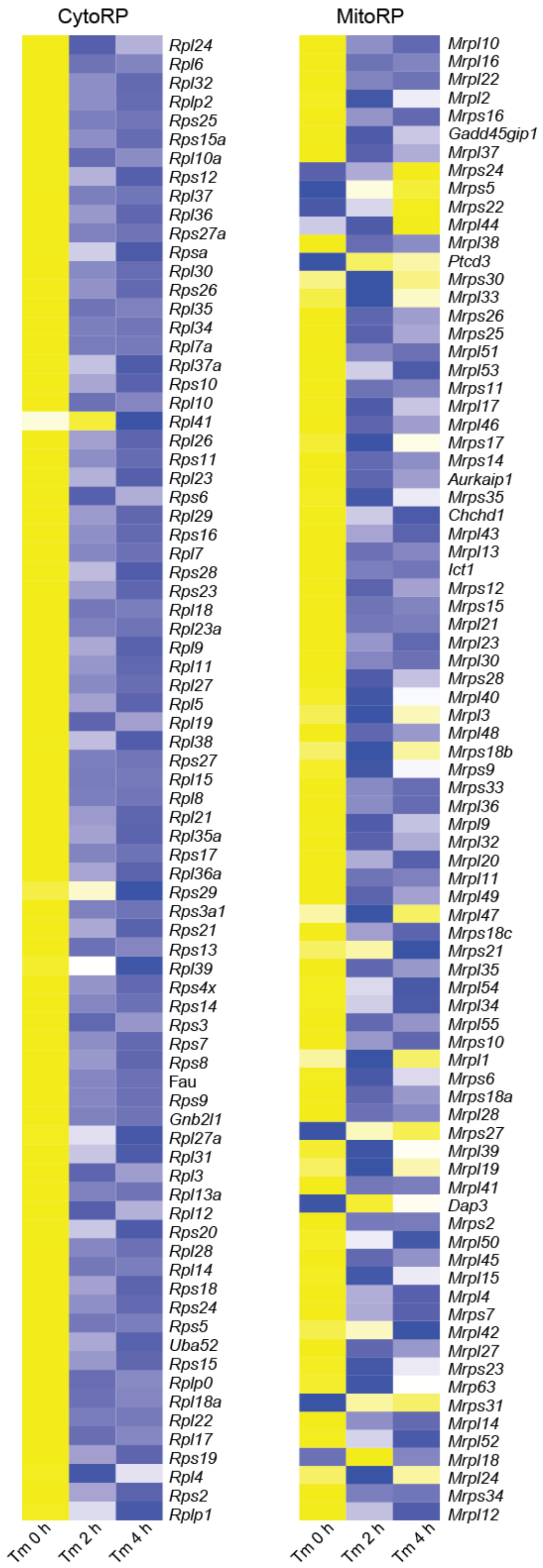

Figure S5. Cytosolic and mitochondrial ribosomal proteins are translationally downregulated by Tm, Related to Figure 3

(A) Heat map showing z-scores of anota2seq translation efficiencies of all 79 cytosolic ribosomal proteins (CytoRP) subunit-encoding mRNAs at 0, 2 and 4 hours following Tm injection.

(B) Same as (A) but with all 82 mitochondrial ribosomal proteins (MitoRP) subunits.

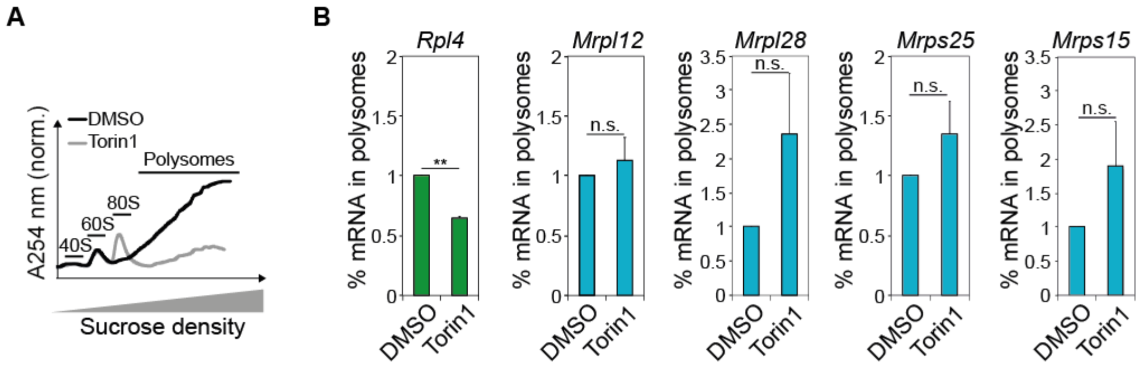

Figure S6. Translation of mitochondrial ribosomal proteins is not sensitive to acute mTORC1 inhibition, Related to Figure 4

(A) Representative polysome profile (optical density of the fractions at 254 nm) on 5-50% sucrose gradients of HeLa cells treated with DMSO or 250 nM of Torin1 for 2 hours.

(B) *Rpl4*, *Mrpl12*, *Mrpl28, Mrps25 and Mrps15* mRNA abundance from heavy polysomal fractions (more than 3 ribosomes) from (A) assessed by qPCR.

Data are means ± SEM (*n* = 5-9). **P<0.01 as determined by the unpaired Student t-test.

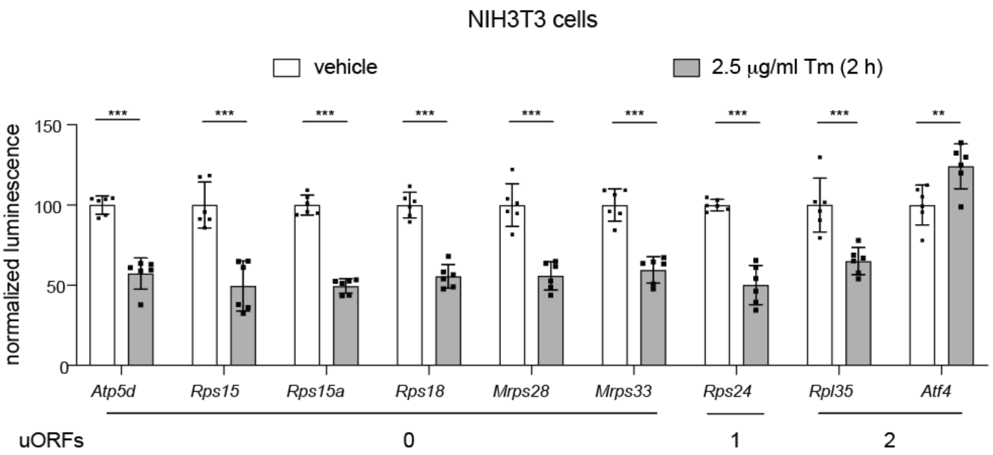

Figure S7. Composition of UTRs regulates the translational response to eIF2α phosphorylation, Related to Figure 5

Firefly luciferase activity in NIH3T3 cells transfected with reporter mRNAs containing the 5′UTRs of indicated mRNAs and treated with either vehicle or 2.5 μg/ml Tunicamycin (Tm) for 2 hours. Firefly luciferase activity was normalized against a transfection control (see Methods) and vehicle-treated cells. Data are means ± SEM (n=6). Statistical analysis was carried out in Prism 8 using a two-way ANOVA test.

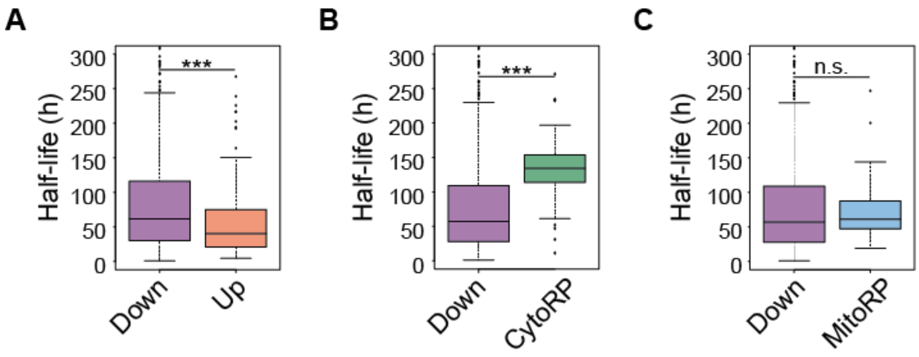

Figure S8. Translationally downregulated mRNAs encode for highly stable proteins, Related to Figure 6

Same as in Figure 6E with protein half-lives analyzed from (Schwanhäusser et al, 2013).

(A) Boxplot of half-lives of proteins encoded by down- or upregulated (Down or Up) mRNAs at 2 hours following Tm injection.

(B) Same as (A) comparing the half-lives of proteins encoded by translationally downregulated mRNAs with all 79 CytoRP subunits 2 hours after Tm.

(C) Same as (B) with all 82 MitoRP subunits. (A-C) ***P<0.001 as determined by the unpaired Wilcoxon test.

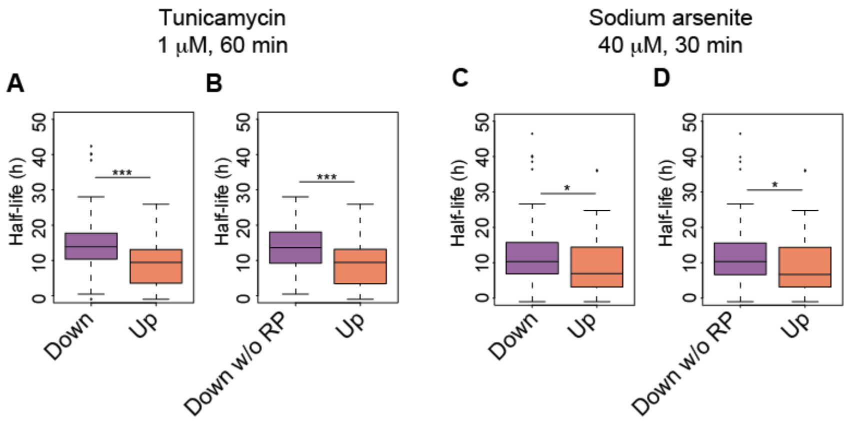

Figure S9. eIF2α phosphorylation induced by different stressors downregulates translation of mRNAs encoding for stable proteins, Related to Figure 6

(A) Boxplot of half-lives of proteins encoded by the translationally down or upregulated (Down or Up) mRNAs using data from (Sidrauski et al, 2015) 1 hours following Tm treatment of HEK293T cells.

(B) Same as (A) but excluding the RPs from the category of translationally downregulated mRNAs (Down w/o RP).

(C) Same as (A) but with data from (Andreev et al, 2015) acquired 30 minutes following arsenite treatment of HEK293T cells.

(D) Same as (C) but excluding the RPs from the category of translationally downregulated mRNAs (Down w/o RP).

(A-D) Protein half-lives were analyzed from (Ly et al, 2018)

*P<0.05, ***P<0.001 as determined by the unpaired Wilcoxon test.
